# Supplementary material for: Silver Clinic: protocol for a feasibility randomised controlled trial of comprehensive geriatric assessment for people living with HIV and frailty
Source: BMJ Open. 2023 May 18;13(5):e070590. doi: 10.1136/bmjopen-2022-070590 (PMC10201220; doi:10.1136/bmjopen-2022-070590)
Supplement: Supplementary data [file bmjopen-2022-070590supp001.pdf]

UNIVERSITY OF SUSSEX

NHS
University Hospitals Sussex
NHS Foundation Trust

CONSENT FORM FOR PROJECT PARTICIPANTS

Title of Project: Feasibility and acceptability of case-finding and subsequent comprehensive geriatric assessment intervention for older people with HIV comprehensive geriatric assessment intervention for older people with HIV

Name of Principal Investigator: Dr Jaime Vera

Health Research Authority Ethics Committee. Ref No: 21/EM/0200

Please state yes or no:

1 I confirm that I have read and understood the information sheet dated 15/11/2021 (version 4). I have had the chance to ask questions about the study and am satisfied with the answers I have been given.

2 I understand that my participation is voluntary and that I am free to withdraw at any time without giving any reason, without my medical care or legal rights being affected.

3 I understand that relevant sections of my medical notes and data collected during the study may be looked at by researchers from Brighton & Sussex Medical School, from regulatory authorities or from the NHS Trust, where it is relevant to my taking part in this research. I give permission for these individuals to have access to my records.

4 I am happy to be contacted about being involved in a one-to-one interview during the study period (optional).

5 If I choose to take part in the interview, I consent to being interviewed by the researcher (only applicable if YES to No 4).

6 I agree to allowing the interview to be audio recorded and the possible use of quotes, that have been anonymised so that I cannot be identified, to be used in any written study reports. (only applicable if YES to No 4).

7 I consent to the processing of my personal information and data for the purposes of this research study. I understand that such information will be treated as confidential and handled in accordance with data protection legislation.

8 I agree to my GP being informed of my participation in the study.

9 I agree to my medical records being accessed for the purposes of this research study.

Consent form- IRAS Project ID: **300599**
V3 – The silver clinic study
14/09/2021

UNIVERSITY OF SUSSEX

NHS University Hospitals Sussex NHS Foundation Trust

10 I understand that the information held and maintained by University Hospitals Sussex may be used to help contact me or provide information about my health status.

11 I wish to receive a summary of the study results.

12 I would like to receive a summary of the findings of the study when they have been published by email or by post (please insert either email or postal address below).

Email:

Address:

13 I consent to take part in the above study.

___________________________	__________________	________________________
Name of Participant	Date	Signature
___________________________	__________________	________________________
Name of Researcher<br>or Person Seeking Consent<br>(If different from researcher)	Date	Signature

When completed: 1 copy for the participant; 1 copy for the researcher site file; *1 copy (original) to be kept in medical notes*

Consent form- IRAS Project ID: **300599**
V3 – The silver clinic study
14/09/2021
