# Supplementary material for: Silver Clinic: protocol for a feasibility randomised controlled trial of comprehensive geriatric assessment for people living with HIV and frailty
Source: BMJ Open. 2023 May 18;13(5):e070590. doi: 10.1136/bmjopen-2022-070590 (PMC10201220; doi:10.1136/bmjopen-2022-070590)
Supplement: Author's manuscript [file bmjopen-2022-070590.draft_revisions.pdf]

BMJ Open

BMJ Open is committed to open peer review. As part of this commitment we make the peer review history of every article we publish publicly available.

When an article is published we post the peer reviewers' comments and the authors' responses online. We also post the versions of the paper that were used during peer review. These are the versions that the peer review comments apply to.

The versions of the paper that follow are the versions that were submitted during the peer review process. They are not the versions of record or the final published versions. They should not be cited or distributed as the published version of this manuscript.

BMJ Open is an open access journal and the full, final, typeset and author-corrected version of record of the manuscript is available on our site with no access controls, subscription charges or pay-per-view fees (http://bmjopen.bmj.com).

If you have any questions on BMJ Open's open peer review process please email info.bmjopen@bmj.com

BMJ Open

The Silver Clinic: protocol for a feasibility randomised controlled trial of Comprehensive Geriatric Assessment for people living with HIV and frailty

Journal:	*BMJ Open*
Manuscript ID	bmjopen-2022-070590
Article Type:	Protocol
Date Submitted by the Author:	29-Nov-2022
Complete List of Authors:	St. Clair-Sullivan, Natalie; Brighton and Sussex Medical School; King's College London, Cicely Saunders Institute of Palliative Care, Policy & Rehabilitation<br>Bristowe, Katherine; King's College London, Cicely Saunders Institute, Department of Palliative Care, Policy and Rehabilitation<br>Adler, Zoe; University Hospitals Sussex NHS Trust<br>Bremner, Stephen; Brighton and Sussex Medical School<br>Harding, Richard; King's College London, Cicely Saunders Institute, Department of Palliative Care, Policy and Rehabilitation<br>Levett, Thomas; Brighton and Sussex Medical School<br>Maddocks, Matthew; King's College London, Cicely Saunders Institute of Palliative Care, Policy & Rehabilitation<br>Pargeter, Gary; Lunch Positive<br>Roberts, Jonathan; University Hospitals Sussex NHS Trust<br>Yi, Deokhee; King's College London, Department of Palliative Care, Policy and Rehabilitation<br>Vera, Jaime; Brighton and Sussex Medical School
Keywords:	HIV & AIDS < INFECTIOUS DISEASES, GERIATRIC MEDICINE, PRIMARY CARE, QUALITATIVE RESEARCH

SCHOLARONE™
Manuscripts

The Silver Clinic: protocol for a feasibility randomised controlled trial of Comprehensive Geriatric Assessment for people living with HIV and frailty

Natalie St Clair-Sullivan[1,2], Katherine Bristowe[2], Zoe Adler[3], Stephen Bremner[1], Richard Harding[2], Thomas Levett[1], Matthew Maddocks[2], Gary Pargeter[4], Jonathan Roberts[3], Deokhee Yi[2], Jaime Vera[1]

[1]Brighton and Sussex Medical School, Brighton, East Sussex, UK; [2]King's College London, Cicely Saunders Institute of Palliative Care, Policy & Rehabilitation, London, UK; [3]University Hospitals Sussex NHS Trust, Brighton, UK; [4]Lunch Positive, Brighton, UK

Corresponding Author:

Natalie St Clair-Sullivan; N.StClair-Sullivan@bsms.ac.uk Brighton and Sussex Medical School, Brighton, East Sussex BN1 9PX

Trial registration number

ISRCTN14646435

Protocol version 6, 10/08/2022

Word count: 3035

Keywords: HIV, Frail Elderly, Geriatric Assessment, integrated care, trial protocols, feasibility Studies, mixed-methods

For peer review only - http://bmjopen.bmj.com/site/about/guidelines.xhtml

ABSTRACT

Introduction

Many people ageing with HIV are also living with multiple comorbidities and geriatric syndromes including frailty and cognitive deterioration. These complex needs can be challenging to meet within existing HIV care services. This study investigates the acceptability and feasibility of screening for frailty and of using a comprehensive geriatric assessment approach, delivered via the Silver Clinic, to support people living with HIV affected by frailty.

Methods and analysis

Mixed-methods, multicentre, parallel-group, randomised, controlled feasibility trial aiming to recruit 84 people living with HIV ≥50, identified as frail. Participants will be randomised 1:1 to receive usual HIV care or the Silver clinic intervention, which uses a Comprehensive Geriatric Assessment approach. Psychosocial, physical and service use outcomes will be measured at baseline, 26 weeks and 52 weeks. Qualitative interviews will be conducted with a subset of participants from both arms. Primary outcome measures include recruitment and retention rates and completion of clinical outcome measures. These will be used in conjunction with a priori progression criteria and the qualitative data (acceptability of trial procedures and intervention) to determine the feasibility and design of a definitive trial.

Ethics and dissemination

This study has been approved by East Midlands – Leicester Central Research Ethics Committee (reference 21/EM/0200). Results will be disseminated via peer-reviewed journals, conferences, and community engagement.

For peer review only

Article Summary

Strengths and limitations of this study
• This is the first study to evaluate the feasibility of screening for frailty and applying a comprehensive geriatric assessment, delivered via outpatient HIV services.<br>• Case finding for frailty could provide a useful tool to guide the delivery of Comprehensive Geriatric Assessment oriented care for people with HIV and frailty that are currently unable to access geriatric services due to younger chronological age.<br>• This study will provide evidence for the implementation of models of care for people living with HIV and affected by frailty.<br>• Integrated geriatric and HIV care may improve healthcare experiences for people living with HIV.<br>• It is not possible to blind participants to their trial arm or the healthcare professionals delivering the intervention, however the healthcare professionals will not know which individuals seen in the Silver Clinic intervention are trial participants.

INTRODUCTION

Of the people accessing HIV services in the UK, 39% are now aged 50 and over (1,2). People living with HIV (PLWH) over 50 appear to experience a disproportionate amount of comorbidities in comparison to their HIV negative counterparts, particularly in regard to geriatric syndromes, such as frailty and cognitive deterioration, which they experience at younger ages (3,4). Studies including a younger cohort of PLWH aged 50-64 demonstrate a frailty prevalence comparable to that of HIV negative cohorts aged 65 and older (5). As such, PLWH may not yet have reached the current UK recommended ages for frailty identification advocated in primary, secondary and community care settings (6,7). This coupled with the potential limited access to geriatric and other frailty services based on age alone, runs the risk of delayed identification of frailty, and identification at a more severe stage where interventions may be less effective, resulting in greater health and social care costs (8).

Current models of HIV care are not addressing the needs of people with HIV, with 47% of health care and 62% of social care needs not being met (9). Moreover, current care models may disadvantage older people living with HIV (OPWH) with, or at risk of, frailty as they can bounce between specialist HIV services and primary care. HIV specialist healthcare professionals (HCPs) often lack the awareness and experience to identify and manage frailty, and many GPs lack knowledge and confidence around HIV (10,11). Use of multiple services can be especially challenging for some OPWH who avoid seeking care in non-HIV services because of perceived or experienced stigma and discrimination (12), which is often highlighted in community engagement work (13–15).

To address this problem the British HIV Association (BHIVA) standards of Care for PLWH state that involvement of a geriatrician with HIV knowledge will strengthen service provision, though how to

For peer review only - http://bmjopen.bmj.com/site/about/guidelines.xhtml

achieve this is unknown (16). The European AIDS Clinical Society (EACS) guidelines recommend screening for frailty in people with HIV (17) and whilst tools to identify patients at risk of frailty using scoring methods are increasingly used internationally (18) and have recently been integrated into UK primary care, it is unknown if screening for frailty among OPWH is acceptable, feasible and useful as part of HIV services, particularly for those who are not chronologically elderly. Evidence-based models of care for OPWH at risk of frailty are needed to inform services on how to best to provide care for patients as described by The King's Fund: The future of HIV services in England, shaping the response to changing needs document (19). Two national surveys led by our team (20,21) and work by community organisations (4) underscore the need for evidence-based guidance on how to best to provide care for OPWH.

The Comprehensive Geriatric Assessment (CGA) is a multidimensional, interdisciplinary diagnostic process used to determine the medical, psychosocial, and functional capabilities of older adults. The CGA has been studied both as a hospital-based programme and as an outpatient consultative service (22) (integrated or separate) to other subspecialties of medicine such as haematology (23), nephrology (24), and oncology (25), and in multimorbidity (26) where evidence suggests that screening for frailty and delivering CGA-based care can improve treatment decision making and reduce risk of institutionalisation when applied to other chronic conditions (27,28). Meta-analyses have demonstrated that CGA in older HIV-negative individuals can delay the development of disability, reduce admissions and hospital stays, and improve survival and functional ability (27–29). However, it is not clear whether CGA can improve outcomes for those OPWH with frailty.

There are few geriatric clinics for people with HIV with published data (30–33); most are ageing clinics set up in Europe and the USA, with different objectives according to local circumstances which lack robust evaluation. Therefore, this will be the first study to evaluate the feasibility of screening for frailty and applying the CGA, delivered through a joint HIV-ageing clinic (the 'Silver Clinic') in outpatient HIV services. Our findings can inform the implementation of models of care for PLWH at risk of frailty.

OBJECTIVES

The aim of this study is to assess the feasibility and acceptability of screening for frailty in OPWH and the Silver Clinic intervention, using a CGA approach; and to test the feasibility of a randomised controlled trial (RCT) to evaluate this intervention in the wider HIV setting. The main objectives are (1) to determine a sample size and primary outcome for a definitive RCT, and (2) to explore what frailty means, what outcomes matter and the experience of the trial processes for OPWH, including communication about the trial, recruitment, randomisation, completion of measures, and experience

For peer review only - http://bmjopen.bmj.com/site/about/guidelines.xhtml

of participation in the trial. Secondary objectives are to (3) to undertake preliminary cost/service utilization analysis and establish cost analysis outcomes for a definitive trial; (4) evaluate the feasibility and acceptability of implementing frailty screening and the Silver Clinic as part of HIV care; (5) To identify development needs and changes required to optimize the referral pathways, clinic structures and the intervention in preparation for a definitive trial; (6) To explore the acceptability of measures of frailty for OPWH. The objectives of the health economic analysis are: 1) to estimate the costs of the intervention; 2) to understand and estimate the costs of formal health and social care and informal care among patients with HIV and frailty; 3) to examine the feasibility of conducting cost-effectiveness analysis of this intervention in the full trial

METHODS AND ANALYSIS

Trial design

The Silver Clinic feasibility study will use a mixed-method randomised controlled trial design. Participants will be randomised 1:1 to two parallel groups: usual care, or the Silver Clinic intervention, (including the CGA). Quantitative data (including process data and participant outcome measures at baseline, week 26 and week 52) will be collected alongside nested qualitative interview data from a subset of participants.

Setting

Participants will be recruited from the HIV unit at the Royal Sussex County Hospital (RSCH), University Hospitals Sussex NHS Foundation Trust (UHSx), Brighton, UK. Data collection will take place at either the RSCH where participants receive their usual HIV care or at the Clinical Research Facility, RSCH.

Eligibility criteria

Inclusion and exclusion criteria are shown in Table 1

Table 1 Inclusion and exclusion criteria

Inclusion criteria	Exclusion criteria
PLWH aged 50 years or older with evidence of frailty scoring 3+ on frailty screening, using the FRAIL scale (34)	PLWH aged under 50 or not defined as frail
Consent to contact the GP	Attended the Silver Clinic during the last 12 months

For peer review only - http://bmjopen.bmj.com/site/about/guidelines.xhtml

Patient and public involvement

A patient and public representative is a named co-applicant on this study (GP) and is the HIV representative of Community Works for all voluntary HIV organisations. Community Works is the largest network of voluntary organisations in Sussex. They are also the manager of Lunch Positive, a weekly lunch club for people living with HIV providing a community space where OPWH in particular have the opportunity to socialise and access HIV peer support. They will chair the Dissemination Working Group of patient and public involvement and community representatives. Additional PPI representatives recruited during the trial will sit on the dissemination working group. They will be actively involved in the development of the study resources, impact and dissemination strategy and all associated activities. The group will have input on study design and recruitment strategies, review of participant facing materials, input into study conduct, monitoring, evaluation, and dissemination of results to participants, service users, community, and national HIV organisations.

Trial procedures

The schedule of assessments is summarised in table 2

Table 2 Summary of trial procedures

**Study visit day**	**Within 4 weeks Baseline**	**Day 0**	**Week 26** +/- 14days	**Week 52** (12 months) +/- 14 days
**Description of visit**	**Screening**	**Baseline**	**First follow up**	**Final visit**
Informed consent	X			
Review eligibility	X	X		
Demographic data	X			
Antiretroviral/medical history	X	X	X	X
Healthcare utilisation data[1]		X	X	X
Silver Clinic Consultation (intervention arm only)		X	X	X
Usual care (control arm)		X	X	X
Frailty measures[2]		X	X	X
PRO[3]		X	X	X

[1] Number of referrals to primary, secondary and social care

[2] Fried frailty phenotype measure, FRAIL scale, Timed up and go test, Rockwood clinical frailty scale, Montreal Cognitive Assessment (MoCA)

[3] Patient reported outcomes as HIV PROM, EuroQol, Adult Social Care Outcomes Toolkit (ASCOT), Client Service Receipt inventory (CSRI), Consultation and Relational Empathy (CARE)

For peer review only - http://bmjopen.bmj.com/site/about/guidelines.xhtml

Recruitment

84 participants will be recruited from the Royal Sussex County Hospital. Potentially eligible individuals will be identified at their routine HIV annual health check attending the Lawson Unit in Brighton. The HIV annual health check takes place for all service users as part of usual care. The health check is performed by nurses and includes assessment of weight, blood pressure, urinalysis, mental health assessment, sexual health screening, adherence review and cervical cytology and contraception. During this assessment, patients will be screened for frailty using the FRAIL Scale (34).

Those expressing interest will be put in contact with the research assistant or nurse to explain the full details of the study, answer any questions, and to give informed consent. Participants will be consecutively enrolled during the period of recruitment, which is anticipated to continue for 12 months.

Interventions

Usual care

Participants allocated to the control arm will receive healthcare from their HIV physician, GP and community services as standard (see Figure 1). HIV standard of care is provided twice a year and most primary care is provided when actively sought by patients. Participants will be aware of their frailty status, provided with an information leaflet about frailty and will consent to the sharing of the result of their frailty assessment with their GP and HIV physician. Participants will be provided with generic healthy ageing advice but will have no access to the intervention. At the end of 12-months, all control participants, who on assessment continue to require specialist input from ageing experts, will be offered the opportunity to attend the Silver Clinic.

The Silver Clinic

Intervention participants will be reviewed within the Silver Clinic based within the Lawson unit, HIV-service at RSCH. The intervention consists of a CGA approach delivered in a joint HIV geriatrics clinic, providing multidisciplinary assessment and management of geriatric syndromes affecting OPWH including frailty, falls, polypharmacy, multimorbidity, and medication-related problems associated with antiretroviral therapy (ART) (see Figure 1). It also supports OPWH with social and psychological challenges, by formulating health interventions such as physical activity and peer support. The appointment consists of patient history taking, physical examination, blood sample and review of medications, cognition, social and mental health. An individualised care plan will then be generated and sent to the patient's GP/HIV physician. The clinic is delivered once a month with individual

For peer review only - http://bmjopen.bmj.com/site/about/guidelines.xhtml

appointments of 40 minutes duration for a total of 16 patients per month. Follow-up frequency in the study will include visits at 6 and 12 months.

Figure 1 Usual HIV care vs the Silver Clinic intervention

Feasibility outcomes and progression criteria

Primary outcomes: To determine whether a definitive trial is feasible, we will examine the recruitment rates, completion rates of study outcome measures, and retention at specific time points. A priori criteria for trial feasibility and progression to full trial without changes to the trial design are as follows (see table 3 For further details):

- Recruitment of 60% of eligible patients;
- Recruitment of 84 patients within 6 months; from first patient randomised;
- Retention of 70 participants (allowing up to 15% attrition) to primary end point (6 months);
- Outcome measure completion for 90% of available participants at each time point.

Secondary outcomes: Health service utilisation (CSRI), social care (ASCOT) and physical and mental health components of the HIV PROM and EuroQol index score and visual analogue scale at 6 and 12 months. In addition, satisfaction with care will also be measured at each timepoint using the CARE measure.

For peer review only

For peer review only - http://bmjopen.bmj.com/site/about/guidelines.xhtml

Objective.	Feasibility outcomes	Contributing data	Progression criteria<br>Green	Amber	Red
1	*Identification & recruitment of eligible patients	Screening & recruitment log	≥60% eligible recruited	59-40%	<40%
3	*Retention of participants at follow up	Participation data	≥70 pts at 3 months<br>≥55 pts at 6 months	74-60%<br>59-40%	<60%<br><40%
4	Contamination of the control arm	CGA service data	≥10% participants receive a CGA within usual care	≥11-20%	≥20%
5	*Outcome measure completion	Participation data	Missing data of ≤10% for each measure.<br>Participant-reported acceptability.	11-25%<br>Some	>25%<br>None
6	Participant satisfaction with care	Participant questionnaires & interviews	Reported as acceptable (or can be with minimal modification)	Reported as acceptable with modification	Intervention not acceptable

Table 3: Silver study feasibility outcomes, contributing data and progression criteria

***primary focus; Traffic-light progression criteria**(35,36) **- Green: likely no concerning issues, Amber: potentially remediable issues , Red: potentially intractable issues**

Data collection

Baseline demographic data will be collected including personal characteristics (age, gender, sex, ethnicity) and social factors (marital status, residential status, formal education level) and comorbidities. Demographic data and patient record data capture for the enrolment and follow-up forms will be done by manual data keying or electronically. Manual data keying is performed in a secure online browser-based platform called REDCap. Electronic data capture entails local extraction of data from clinical electronic databases and will be stored securely on the UHSx systems, HIV drive. Only the research team (including research administrator) will have access to this data and will not be made available outside the team or institution.

Process data will be collected to understand intervention delivery and trial design appropriateness. For the Silver Clinic intervention, records pertaining to CGA date, recommendations and follow up will be collected. For trial process data, trial screening, recruitment rates, participation at each timepoint and amount of missing data will be recorded.

For peer review only - http://bmjopen.bmj.com/site/about/guidelines.xhtml

Standardised clinical outcome measures that represent multiple health and healthcare service domains will be collected at baseline, week 26 and week 52 post randomisation. The Positive Outcomes HIV PROM measures multidimensional symptoms and concerns for PLWH (37,38); the EuroQol EQ-5D-5L measures health related quality of life (39); the Adult Social Care Outcomes Toolkit (ASCOT) measures Social care related quality of life (40); the Client Service Receipt Inventory (CSRI) measures services and support accessed (41); the Consultation and relational empathy measure (CARE) is used to assess interpersonal quality of healthcare encounters (42); the Fried Frailty Phenotype and FRAIL Scale are used to assess physical frailty (34,43); and the Timed Up and Go test to assess functional mobility and falls risk (44). Physical tests will be conducted by the researcher and questionnaires will be completed with support of the researcher.

Nested qualitative interviews

OPWH will be recruited for qualitative interview via purposive sampling from within the RCT participants. OPWH will be purposively sampled by trial arm, age, gender, duration of HIV diagnosis, ethnicity, sexual orientation, living situation and frailty score, to ensure a maximum variation sample.

A purposive sample of up to 15 participants from each arm of the trial will be interviewed on completion of trial participation to examine experiences of: recruitment to the trial, management of their priority concerns during the course of the trial, referral to the Silver Clinic, the description of CGA, experience of the Silver Clinic and perceived impact upon priority outcomes (intervention arm only), satisfaction with care and acceptability of participating in an RCT of the Silver Clinic intervention for OPWH. Draft topic guides will be reviewed by PPI members. Face-to-face, telephone or video call interviews will be conducted by the research assistant and take place in a location of the participant's choosing. Interviews will be digitally audio recorded. Field notes will be used to record contextual factors, participant responses, and personal reflections.

Sample size

This is a feasibility trial and therefore not powered to test effectiveness of the intervention compared to standard treatment. However, data will be used to inform the sample size calculation for a future definitive trial. To estimate the parameters needed for a future trial with sufficient precision, a sample size of at least 35 participants per arm is recommended(45,46). Based on the local patient numbers (cohort of 2450; 54% over 50 years old), we anticipate recruiting 42 patients per arm i.e. 84 in all allowing for attrition of 15% to be achievable.

Randomisation and blinding

For peer review only - http://bmjopen.bmj.com/site/about/guidelines.xhtml

For peer review only

After the baseline assessment the research assistant will randomise participants using REDCap in a 1:1 allocation to receive either usual care (Control arm) or referral to the Silver Clinic (Intervention arm), stratifying one age (50-56, 66-80, 81-95, 96-110) and sex to ensure a balanced sample in both arms. Clinicians delivering the intervention will be blinded to the screening and randomisation process as they will have no knowledge of when patients are screened for frailty nor have any influence on the randomisation process, minimising the possibility of selection bias.

Analysis

Quantitative data

Baseline characteristics of the intervention and control participants will be summarised using descriptive statistics. Participant flow through the trial will be shown on a flowchart according to the CONSORT 2010 Statement extension for pilot and feasibility trials (47). Data will be presented by trial arm. Normally distributed variables will be summarised by their means and standard deviations, skewed continuous variables by their medians and interquartile ranges and categorical variables by their frequencies and percentages. For the feasibility outcomes, proportion of patients recruited, participants retained and data completeness 95% confidence intervals will be presented. Differences in means between arms for the secondary outcomes will be presented with 95% confidence intervals. Analysis will be of available cases following intention to treat principles. Missing data will be quantified but not imputed. A full statistical analysis plan will be agreed prior to database lock for the final analysis.

Qualitative data

Interview and focus group recordings will be transcribed verbatim and pseudonymised (removing any identifiable characteristics). Interviews will be analysed using reflexive thematic analysis, in six stages: familiarisation; coding; searching; reviewing; defining themes; and reporting (48,49). Analysis will be reviewed by the study team, including PPI members, and revisited to develop a theoretical model of person-centred care for OPWH and frailty. Analysis will be supported using NVivo qualitative data analysis software, and reported in accordance with the consolidated criteria for reporting qualitative studies (COREQ) (50).

Cost analysis

Data for costs will be collected by the modified Client Service Receipt Inventory (CSRI), which asks patients about the health and social care service use and informal care provided by family and/or friends. Response rate for EQ-5D-5L and visual analog scale at different time points will be checked and described. EQ-5D index scores will be calculated using the Crosswalk value set using EQ-5D-3L in

For peer review only - http://bmjopen.bmj.com/site/about/guidelines.xhtml

the UK as recommended by the NICE(51). We will examine the completion rates for each item in outcome measurements and CSRI first. Unit costs for each service item will be collected from usual data sources (e.g. NHS Reference costs, PSSRU Unit costs of health and social care, market wage rates). Then, we will describe and compare the utilization and costs of formal care (health and social care) and informal care provided by family/friends. Costs of caring for patients in this group are of interest to commissioning purposes. We will describe the patterns of service uses and costs by types of services (e.g. acute care, community care) at different time points. The intervention costs will be estimated using records from trial management teams and CSRI. We will try calculating incremental cost-effectiveness ratios (ICERs) of this intervention although we do not aim to use the results to justify the cost-effectiveness of the intervention. We will also explore the uncertainties around the parameters and draw the cost-effectiveness planes using bootstrapping. Because this is the feasibility RCT, we will not be able to make a conclusive remark on cost-effectiveness of the intervention.

Health economic analysis from an NHS perspective uses formal care costs but formal and informal care costs will be used for analysis from a wider societal perspective. We have also included questions about the changes in labour market activities (e.g. stopping working or reducing hours of working due to illness) to investigate the feasibility of including social costs in future economic analysis.

ETHICS AND DISSEMINATION

This study has been approved by East Midlands – Leicester Central Research Ethics Committee (REC reference: 21/EM/0200). Protocol amendments will be communicated to all relevant parties. The findings from this study, positive, negative or inconclusive, are intended to be published in peer-reviewed journals and/or presented at conferences and seminars and disseminated through HIV community groups.

Competing interests

None declared

Funding

This project is funded by the National Institute for Health and Care Research (NIHR) under its Research for Patient Benefit (RfPB) Programme (Grant Reference Number NIHR201060). The views expressed are those of the author(s) and not necessarily those of the NIHR or the Department of Health and Social Care. MM is funded by a National Institute for Health and Care Research (NIHR) Career Development Fellowship (CDF-2017–10-009) and the NIHR Applied Research Collaboration South London (NIHR ARC South London) at King's College Hospital NHS Foundation Trust.

For peer review only - http://bmjopen.bmj.com/site/about/guidelines.xhtml

Author Contributions

KB and JV led on the design of the study. NS drafted the protocol and all authors contributed to the study design, writing, planning and revising the protocol, including the safety assessments. SB wrote the statistical section and analysis plan and DY wrote the cost analysis section. GP, PPI member, helped develop trial related materials such as the PIS and interview topic guides and contributed to the dissemination strategy. All authors contributed to and approved the final published version of the trial protocol.

Trial sponsor

Trial Sponsor: University of Sussex

Sponsor's Reference: 087 VER/300599

Contact name: Anthony Walsh

Address: Senior Research Ethics & Integrity Officer / Research and Enterprise Services 1st Floor, Falmer House, University of Sussex, Brighton BN1 9QF, United Kingdom

Telephone: +44 (0)1273 872748

Email: researchsponsorship@sussex.ac.uk

For peer review only

References

1. Levett T, Alford K, Roberts J, Adler Z, Wright J, Vera JH. Evaluation of a combined HIV and geriatrics clinic for older people living with HIV: the Silver clinic in Brighton, UK. Geriatrics [Internet]. 2020 [cited 2020 Dec 1];5(81). Available from: http://sro.sussex.ac.uk

2. Piot P, Abdool Karim SS, Hecht R, Legido-Quigley H, Buse K, Stover J, et al. Defeating AIDS - Advancing global health. Lancet. 2015;386(9989):171–218.

3. Bloch M. Frailty in people living with HIV. AIDS Res Ther [Internet]. 2018 Dec 16 [cited 2020 Mar 30];15(1):19. Available from: https://aidsrestherapy.biomedcentral.com/articles/10.1186/s12981-018-0210-2

4. Terrence Higgins Trust. Uncharted Territory A report into the first generation growing older with HIV. 2017.

5. Levett TJ, Cresswell F V., Malik MA, Fisher M, Wright J. Systematic Review of Prevalence and Predictors of Frailty in Individuals with Human Immunodeficiency Virus. J Am Geriatr Soc [Internet]. 2016 May 1 [cited 2021 May 11];64(5):1006–14. Available from: https://pubmed.ncbi.nlm.nih.gov/27225356/

6. Vernon MJ. NHS RightCare: Frailty Toolkit. 2019.

7. British Geriatrics Society. Fit for Frailty Part 1 [Internet]. 2017 [cited 2021 May 14]. Available from: https://www.bgs.org.uk/sites/default/files/content/resources/files/2018-05-23/fff_full.pdf

8. Lundqvist M, Alwin J, Henriksson M, Husberg M, Carlsson P, Ekdahl AW. Cost-effectiveness of comprehensive geriatric assessment at an ambulatory geriatric unit based on the AGe-FIT trial. BMC Geriatr [Internet]. 2018 Jan 31 [cited 2021 May 14];18(1). Available from: https://pubmed.ncbi.nlm.nih.gov/29386007/

9. Public Health England. Positive Voices: The National Survey of People Living with HIV Findings from the 2017 survey [Internet]. 2017 [cited 2021 May 11]. Available from: moz-extension://eacf2752-a225-4ca0-8bae-4f106db5688e/enhanced-reader.html?openApp&pdf=https%3A%2F%2Fassets.publishing.service.gov.uk%2Fgovernment%2Fuploads%2Fsystem%2Fuploads%2Fattachment_data%2Ffile%2F857922%2FPHE_positive_voices_report_2019.pdf

10. Liddy C, Shoemaker ES, Crowe L, Boucher LM, Rourke SB, Rosenes R, et al. How the delivery of HIV care in Canada aligns with the Chronic Care Model: A qualitative study. PLoS One [Internet]. 2019 Jun 1 [cited 2021 Aug 25];14(7):e0220516. Available from: https://journals.plos.org/plosone/article?id=10.1371/journal.pone.0220516

11. Siegler EL, Burchett CO, Glesby MJ. Older people with HIV are an essential part of the continuum of HIV care. J Int AIDS Soc [Internet]. 2018;21:25188. Available from: http://onlinelibrary.wiley.com/doi/10.1002/jia2.25188/full%7Chttps://doi.org/10.1002/jia2.25188

12. StigmaSurveyUK. HIV in the UK: Changes and Challenges; Actions and Answers The People Living With HIV Stigma Survey UK 2015 Women [Internet]. 2015 [cited 2020 Oct 20]. Available from: http://www.nat.org.uk/media/Files/PDF documents/Mori_2014_report_FINAL.pdf

13. Nixon E, Cooper V, Youssef E, Smith H, Robert G, Fisher M. NH4 Service development for people with HIV who have comorbid conditions using experience-based co-design methodology. Sex Transm Infect [Internet]. 2016 Jun 1 [cited 2021 May 14];92(Suppl 1):A18.1-A18. Available from: https://sti.bmj.com/content/92/Suppl_1/A18.1

For peer review only - http://bmjopen.bmj.com/site/about/guidelines.xhtml

14. Pollard A, Llewellyn C, Cooper V, Sachikonye M, Perry N, Nixon E, et al. Patients' perspectives on the development of HIV services to accommodate ageing with HIV: a qualitative study. Int J STD AIDS [Internet]. 2018 Apr 1 [cited 2021 May 14];29(5):483–90. Available from: https://journals.sagepub.com/doi/abs/10.1177/0956462417735723

15. Miners AH, Llewellyn CD, Cooper VL, Youssef E, Pollard AJ, Lagarde M, et al. A discrete choice experiment to assess people living with HIV's (PLWHIV's) preferences for GP or HIV clinic appointments. Sex Transm Infect [Internet]. 2017 Mar 1 [cited 2021 May 14];93(2):105–11. Available from: http://sti.bmj.com/

16. BHIVA. Standards of Care for People Living with HIV [Internet]. 2018. Available from: https://www.bhiva.org/file/KrfaFqLZRlBhg/BHIVA-Standards-of-Care-2018.pdf

17. European AIDS Clinical Society. European AIDS Clinical Society Guidelines [Internet]. 2019. Available from: https://wwweacsocietyorg/files/2019_guidelines-100_finalpdf

18. Romero-Ortuno R, Soraghan C. A Frailty Instrument for primary care for those aged 75 years or more: Findings from the Survey of Health, Ageing and Retirement in Europe, a longitudinal population-based cohort study (SHARE-FI75+). BMJ Open [Internet]. 2014 Dec 1 [cited 2021 May 11];4(12):6645. Available from: http://bmjopen.bmj.com/

19. Baylis A, Buck D, Anderson J, Jabbal J, Ross S. The future of HIV services in England Shaping the response to changing needs [Internet]. 2017 [cited 2019 Aug 16]. Available from: https://www.kingsfund.org.uk/sites/default/files/field/field_publication_file/Future_HIV_services_England_Kings_Fund_April_2017.pdf

20. Cresswell F, Levett T. Specialist care of older adults with HIV infection in the UK: a service evaluation. HIV Med [Internet]. 2017 Aug 1 [cited 2021 Feb 9];18(7):519–24. Available from: http://doi.wiley.com/10.1111/hiv.12481

21. Youssef E, Wright J, Cooper V, Nixon E, Vera J. Current models of care for the management of HIV patients with comorbidities in England: a survey. J Int AIDS Soc. 2016;19(7).

22. Smith SM, Wallace E, O'Dowd T, Fortin M. Interventions for improving outcomes in patients with multimorbidity in primary care and community settings [Internet]. Vol. 2016, Cochrane Database of Systematic Reviews. John Wiley and Sons Ltd; 2016 [cited 2021 May 11]. Available from: https://pubmed.ncbi.nlm.nih.gov/26976529/

23. Hamaker ME, Mitrovic M, Stauder R. The G8 screening tool detects relevant geriatric impairments and predicts survival in elderly patients with a haematological malignancy. Ann Hematol [Internet]. 2014 [cited 2021 May 11];93(6):1031–40. Available from: https://pubmed.ncbi.nlm.nih.gov/24488257/

24. Kallenberg MH, Kleinveld HA, Dekker FW, van Munster BC, Rabelink TJ, van Buren M, et al. Functional and cognitive impairment, frailty, and adverse health outcomes in older patients reaching ESRD-a systematic review. Clin J Am Soc Nephrol [Internet]. 2016 [cited 2021 May 11];11(9):1624–39. Available from: https://pubmed.ncbi.nlm.nih.gov/27342598/

25. Caillet P, Laurent M, Bastuji-Garin S, Liuu E, Culine S, Lagrange JL, et al. Optimal management of elderly cancer patients: Usefulness of the comprehensive geriatric assessment. Clin Interv Aging [Internet]. 2014 Sep 29 [cited 2021 May 11];9:1645–60. Available from: /pmc/articles/PMC4189720/

26. van Loon IN, Wouters TR, Boereboom FTJ, Bots ML, Verhaar MC, Hamaker ME. The relevance of geriatric impairments in patients starting dialysis: A systematic review. Clin J Am Soc Nephrol [Internet]. 2016 Apr 26 [cited 2021 May 11];11(7):1245–59. Available from:

For peer review only - http://bmjopen.bmj.com/site/about/guidelines.xhtml

https://europepmc.org/articles/PMC4934838

27. Ellis G, Gardner M, Tsiachristas A, Langhorne P, Burke O, Harwood RH, et al. Comprehensive geriatric assessment for older adults admitted to hospital. Cochrane Database Syst Rev [Internet]. 2017 Sep 12 [cited 2021 May 11];(9):1–108. Available from: https://pubmed.ncbi.nlm.nih.gov/28898390/

28. Garrard JW, Cox NJ, Dodds RM, Roberts HC, Sayer AA. Comprehensive geriatric assessment in primary care: a systematic review [Internet]. Vol. 32, Aging Clinical and Experimental Research. Springer; 2020 [cited 2021 Feb 15]. p. 197–205. Available from: https://doi.org/10.1007/s40520-019-01183-w

29. Stuck AE, Iliffe S. Comprehensive geriatric assessment for older adults [Internet]. Vol. 343, BMJ (Online). BMJ; 2011 [cited 2021 May 11]. p. 1029–30. Available from: https://pubmed.ncbi.nlm.nih.gov/22034147/

30. Siegler EL, Burchett CO, Glesby MJ. Older people with HIV are an essential part of the continuum of HIV care [Internet]. Vol. 21, Journal of the International AIDS Society. John Wiley and Sons Inc.; 2018 [cited 2021 May 11]. p. 25188. Available from: /pmc/articles/PMC6178281/

31. Ruiz M, Cefalu C, Ogbuokiri J. A dedicated screening program for geriatric HIV-infected patients integrating HIV and geriatric care. J Int Assoc Physicians AIDS Care [Internet]. 2010 May [cited 2021 May 11];9(3):157–61. Available from: https://pubmed.ncbi.nlm.nih.gov/20530469/

32. Ruiz M, Cefalu C. Characteristics of frail patients in a geriatric-HIV program: The experience of an urban academic center at one year follow-up. J Int Assoc Physicians AIDS Care [Internet]. 2011 May 18 [cited 2021 May 11];10(3):138–43. Available from: http://jiapac.sagepub.com

33. Waters L, Patterson B, Scourfield A, Hughes A, de Silva S, Gazzard B, et al. A dedicated clinic for HIV-positive individuals over 50 years of age: A multidisciplinary experience [Internet]. Vol. 23, International Journal of STD and AIDS. SAGE PublicationsSage UK: London, England; 2012 [cited 2021 May 11]. p. 546–52. Available from: https://journals.sagepub.com/doi/abs/10.1258/ijsa.2012.011412

34. Abellan Van Kan G, Rolland Y, Bergman H, Morley JE, Kritchevsky SB, Vellas B. The I.A.N.A. task force on frailty assessment of older people in clinical practice. J Nutr Heal Aging [Internet]. 2008 Jan [cited 2021 May 14];12(1):29–37. Available from: https://pubmed.ncbi.nlm.nih.gov/18165842/

35. Craig P, Dieppe P, Macintyre S, Michie S, Nazareth I, Petticrew M. Developing and evaluating complex interventions: The new Medical Research Council guidance. Int J Nurs Stud. 2013;50(5):587–92.

36. Brighton LJ, Evans CJ, Farquhar M, Bristowe K, Kata A, Higman J, et al. Integrating Comprehensive Geriatric Assessment for people with COPD and frailty starting pulmonary rehabilitation: the Breathe Plus feasibility trial protocol. ERJ Open Res [Internet]. 2021 Feb 4 [cited 2021 Mar 5];00717–2020. Available from: https://doi.org/10.1183/23120541.00717-2020

37. Bristowe K, Clift P, James R, Josh J, Platt M, Whetham J, et al. Towards person-centred care for people living with HIV : what core outcomes matter, and how might we assess them? A cross-national multi-centre qualitative study with key stakeholders. HIV Med [Internet]. 2019 Jun 4 [cited 2020 Mar 30];20(8):hiv.12758. Available from: https://onlinelibrary.wiley.com/doi/abs/10.1111/hiv.12758

For peer review only - http://bmjopen.bmj.com/site/about/guidelines.xhtml

38. Bristowe K, Murtagh FEM, Clift P, James R, Josh J, Platt M, et al. The development and cognitive testing of the positive outcomes HIV PROM: A brief novel patient-reported outcome measure for adults living with HIV. Health Qual Life Outcomes. 2020;18(1):1–10.

39. Rabin R, De Charro F. EQ-5D: A measure of health status from the EuroQol Group. In: Annals of Medicine [Internet]. Royal Society of Medicine Press Ltd; 2001 [cited 2021 May 14]. p. 337–43. Available from: https://pubmed.ncbi.nlm.nih.gov/11491192/

40. Netten A, Burge P, Malley J, Potoglou D, Towers AM, Brazier J, et al. Outcomes of social care for adults: Developing a preference-weighted measure. Health Technol Assess (Rockv) [Internet]. 2012 [cited 2021 May 14];16(16):1–165. Available from: https://pubmed.ncbi.nlm.nih.gov/22459668/

41. Beecham J, Snell T, Perkins M, Knapp M. Health and social care costs for young adults with epilepsy in the UK. Heal Soc Care Community [Internet]. 2010 Sep [cited 2021 May 14];18(5):465–73. Available from: https://pubmed.ncbi.nlm.nih.gov/20491967/

42. Mercer SW, Maxwell M, Heaney D, Watt GCM. The consultation and relational empathy (CARE) measure: Development and preliminary validation and reliability of an empathy-based consultation process measure. Fam Pract [Internet]. 2004 Dec [cited 2021 May 14];21(6):699–705. Available from: https://pubmed.ncbi.nlm.nih.gov/15528286/

43. Fried LP, Tangen CM, Walston J, Newman AB, Hirsch C, Gottdiener J, et al. Frailty in older adults: Evidence for a phenotype. Journals Gerontol - Ser A Biol Sci Med Sci [Internet]. 2001 [cited 2021 May 11];56(3). Available from: https://pubmed.ncbi.nlm.nih.gov/11253156/

44. Richardson S. The Timed "Up & Go": A Test of Basic Functional Mobility for Frail Elderly Persons. J Am Geriatr Soc [Internet]. 1991 [cited 2021 May 14];39(2):142–8. Available from: https://pubmed.ncbi.nlm.nih.gov/1991946/

45. Sim J, Lewis M. The size of a pilot study for a clinical trial should be calculated in relation to considerations of precision and efficiency. J Clin Epidemiol [Internet]. 2012 Mar 1 [cited 2021 May 14];65(3):301–8. Available from: http://www.jclinepi.com/article/S0895435611002642/fulltext

46. Teare MD, Dimairo M, Shephard N, Hayman A, Whitehead A, Walters SJ. Sample size requirements to estimate key design parameters from external pilot randomised controlled trials: A simulation study. Trials [Internet]. 2014 Jul 3 [cited 2021 May 14];15(1):1–13. Available from: http://www.trialsjournal.com/content/15/1/264

47. Eldridge SM, Chan CL, Campbell MJ, Bond CM, Hopewell S, Thabane L, et al. CONSORT 2010 statement: Extension to randomised pilot and feasibility trials. BMJ [Internet]. 2016 [cited 2021 May 25];355. Available from: https://pubmed.ncbi.nlm.nih.gov/27777223/

48. Braun V, Clarke V. Using thematic analysis in psychology. Qual Res Psychol [Internet]. 2006 [cited 2018 Jul 25];3(2):77–101. Available from: http://www.tandfonline.com/action/journalInformation?journalCode=uqrp20

49. Braun V, Clarke V. Reflecting on reflexive thematic analysis. Qual Res Sport Exerc Heal [Internet]. 2019 Aug 8 [cited 2020 Feb 28];11(4):589–97. Available from: https://www.tandfonline.com/doi/full/10.1080/2159676X.2019.1628806

50. Tong A, Sainsbury P, Craig J. Consolidated criteria for reporting qualitative research (COREQ): A 32-item checklist for interviews and focus groups. Int J Qual Heal Care. 2007;19(6):349–57.

51. National Institute for Health and Care Excellence. Position statement on use of the EQ-5D-5L value set for England (updated October 2019) | Technology appraisal guidance | NICE

For peer review only - http://bmjopen.bmj.com/site/about/guidelines.xhtml

guidance | Our programmes | What we do | About | NICE [Internet]. NICE; 2019 [cited 2022 Nov 18]. Available from: https://www.nice.org.uk/about/what-we-do/our-programmes/nice-guidance/technology-appraisal-guidance/eq-5d-5l

For peer review only

For peer review only - http://bmjopen.bmj.com/site/about/guidelines.xhtml

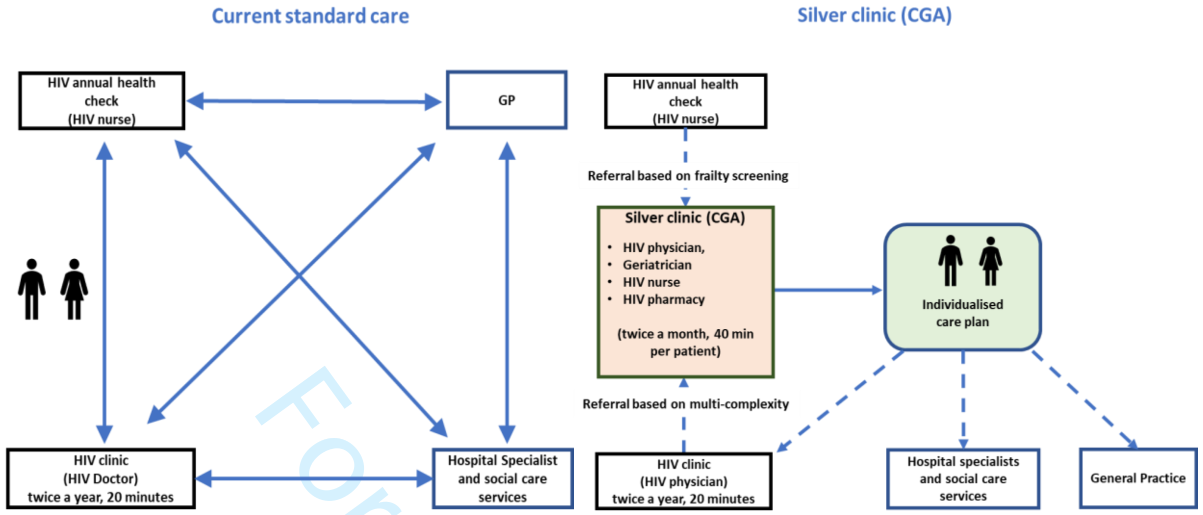

For peer review only

UNIVERSITY OF SUSSEX

University Hospitals Sussex
NHS Foundation Trust

CONSENT FORM FOR PROJECT PARTICIPANTS

Title of Project: Feasibility and acceptability of case-finding and subsequent comprehensive geriatric assessment intervention for older people with HIV comprehensive geriatric assessment intervention for older people with HIV

Name of Principal Investigator: Dr Jaime Vera

Health Research Authority Ethics Committee. Ref No: 21/EM/0200

		Please state yes or no:
1	I confirm that I have read and understood the information sheet dated 15/11/2021 (version 4). I have had the chance to ask questions about the study and am satisfied with the answers I have been given.	
2	I understand that my participation is voluntary and that I am free to withdraw at any time without giving any reason, without my medical care or legal rights being affected.	
3	I understand that relevant sections of my medical notes and data collected during the study may be looked at by researchers from Brighton & Sussex Medical School, from regulatory authorities or from the NHS Trust, where it is relevant to my taking part in this research. I give permission for these individuals to have access to my records.	
4	I am happy to be contacted about being involved in a one-to-one interview during the study period (optional).	
5	If I choose to take part in the interview, I consent to being interviewed by the researcher (only applicable if YES to No 4).	
6	I agree to allowing the interview to be audio recorded and the possible use of quotes, that have been anonymised so that I cannot be identified, to be used in any written study reports. (only applicable if YES to No 4).	
7	I consent to the processing of my personal information and data for the purposes of this research study. I understand that such information will be treated as confidential and handled in accordance with data protection legislation.	
8	I agree to my GP being informed of my participation in the study.	
9	I agree to my medical records being accessed for the purposes of this research study.	

For peer review only - http://bmjopen.bmj.com/site/about/guidelines.xhtml

For peer review only

UNIVERSITY OF SUSSEX

University Hospitals Sussex NHS Foundation Trust

10 I understand that the information held and maintained by University Hospitals Sussex may be used to help contact me or provide information about my health status.

11 I wish to receive a summary of the study results.

12 I would like to receive a summary of the findings of the study when they have been published by email or by post (please insert either email or postal address below).

Email:

Address:

13 I consent to take part in the above study.

Name of Participant	Date	Signature

Name of Researcher or Person Seeking Consent (If different from researcher)	Date	Signature

When completed: 1 copy for the participant; 1 copy for the researcher site file; *1 copy (original) to be kept in medical notes*

For peer review only - http://bmjopen.bmj.com/site/about/guidelines.xhtml

Reporting checklist for protocol of a clinical trial.

Based on the SPIRIT guidelines.

Instructions to authors

Complete this checklist by entering the page numbers from your manuscript where readers will find each of the items listed below.

Your article may not currently address all the items on the checklist. Please modify your text to include the missing information. If you are certain that an item does not apply, please write "n/a" and provide a short explanation.

Upload your completed checklist as an extra file when you submit to a journal.

In your methods section, say that you used the SPIRITreporting guidelines, and cite them as:

Chan A-W, Tetzlaff JM, Gøtzsche PC, Altman DG, Mann H, Berlin J, Dickersin K, Hróbjartsson A, Schulz KF, Parulekar WR, Krleža-Jerić K, Laupacis A, Moher D. SPIRIT 2013 Explanation and Elaboration: Guidance for protocols of clinical trials. BMJ. 2013;346:e7586

		Reporting Item	Page Number
**Administrative information**			
Title	#1	Descriptive title identifying the study design, population, interventions, and, if applicable, trial acronym	1
Trial registration	#2a	Trial identifier and registry name. If not yet registered, name of intended registry	1
Trial registration: data set	#2b	All items from the World Health Organization Trial Registration Data Set	1-13 - Listed throughout and in trial registration
Protocol version	#3	Date and version identifier	1
Funding	#4	Sources and types of financial, material, and other support	12
Roles and responsibilities: contributorship	#5a	Names, affiliations, and roles of protocol contributors	13

For peer review only

Roles and responsibilities: sponsor contact information	#5b	Name and contact information for the trial sponsor	13
Roles and responsibilities: sponsor and funder	#5c	Role of study sponsor and funders, if any, in study design; collection, management, analysis, and interpretation of data; writing of the report; and the decision to submit the report for publication, including whether they will have ultimate authority over any of these activities	N/A - sponsors/funders to not have a role in study activities
Roles and responsibilities: committees	#5d	Composition, roles, and responsibilities of the coordinating centre, steering committee, endpoint adjudication committee, data management team, and other individuals or groups overseeing the trial, if applicable (see Item 21a for data monitoring committee)	6, 9-11
**Introduction**			
Background and rationale	#6a	Description of research question and justification for undertaking the trial, including summary of relevant studies (published and unpublished) examining benefits and harms for each intervention	3-4
Background and rationale: choice of comparators	#6b	Explanation for choice of comparators	3-5
Objectives	#7	Specific objectives or hypotheses	4-5
Trial design	#8	Description of trial design including type of trial (eg, parallel group, crossover, factorial, single group), allocation ratio, and framework (eg, superiority, equivalence, non-inferiority, exploratory)	5
**Methods: Participants, interventions, and outcomes**			

For peer review only

Study setting	#9	Description of study settings (eg, community clinic, academic hospital) and list of countries where data will be collected. Reference to where list of study sites can be obtained	5
Eligibility criteria	#10	Inclusion and exclusion criteria for participants. If applicable, eligibility criteria for study centres and individuals who will perform the interventions (eg, surgeons, psychotherapists)	5, 7-8
Interventions: description	#11a	Interventions for each group with sufficient detail to allow replication, including how and when they will be administered	7-8
Interventions: modifications	#11b	Criteria for discontinuing or modifying allocated interventions for a given trial participant (eg, drug dose change in response to harms, participant request, or improving / worsening disease)	7
Interventions: adherance	#11c	Strategies to improve adherence to intervention protocols, and any procedures for monitoring adherence (eg, drug tablet return; laboratory tests)	N/A - there is nothing for the participants to adhere to apart from attending the Silver Clinic for their appointment, which they will recive a text reminder about.
Interventions: concomitant care	#11d	Relevant concomitant care and interventions that are permitted or prohibited during the trial	7
Outcomes	#12	Primary, secondary, and other outcomes, including the specific measurement variable (eg, systolic blood pressure), analysis metric (eg, change from baseline, final value, time to event), method of aggregation (eg, median, proportion), and time point for each outcome. Explanation of the clinical relevance of chosen efficacy and harm outcomes is strongly recommended	8-9
Participant timeline	#13	Time schedule of enrolment, interventions (including any run-ins and washouts),	6

For peer review only

		assessments, and visits for participants. A schematic diagram is highly recommended (see Figure)	
Sample size	#14	Estimated number of participants needed to achieve study objectives and how it was determined, including clinical and statistical assumptions supporting any sample size calculations	10
Recruitment	#15	Strategies for achieving adequate participant enrolment to reach target sample size	6-7
**Methods: Assignment of interventions (for controlled trials)**			
Allocation: sequence generation	#16a	Method of generating the allocation sequence (eg, computer-generated random numbers), and list of any factors for stratification. To reduce predictability of a random sequence, details of any planned restriction (eg, blocking) should be provided in a separate document that is unavailable to those who enrol participants or assign interventions	11
Allocation concealment mechanism	#16b	Mechanism of implementing the allocation sequence (eg, central telephone; sequentially numbered, opaque, sealed envelopes), describing any steps to conceal the sequence until interventions are assigned	11
Allocation: implementation	#16c	Who will generate the allocation sequence, who will enrol participants, and who will assign participants to interventions	11
Blinding (masking)	#17a	Who will be blinded after assignment to interventions (eg, trial participants, care providers, outcome assessors, data analysts), and how	11
Blinding (masking):	#17b	If blinded, circumstances under which	N/A - there is no change to

For peer review only

emergency unblinding		unblinding is permissible, and procedure for revealing a participant's allocated intervention during the trial	patients HIV care during the trial, therefore if there was an emergency so they would recieve the same care reagrdless of their trial allocation.
**Methods: Data collection, management, and analysis**			
Data collection plan	#18a	Plans for assessment and collection of outcome, baseline, and other trial data, including any related processes to promote data quality (eg, duplicate measurements, training of assessors) and a description of study instruments (eg, questionnaires, laboratory tests) along with their reliability and validity, if known. Reference to where data collection forms can be found, if not in the protocol	9-10
Data collection plan: retention	#18b	Plans to promote participant retention and complete follow-up, including list of any outcome data to be collected for participants who discontinue or deviate from intervention protocols	N/A - as this is a feasibility trial retention and lost to follow-up are things we want to know about
Data management	#19	Plans for data entry, coding, security, and storage, including any related processes to promote data quality (eg, double data entry; range checks for data values). Reference to where details of data management procedures can be found, if not in the protocol	9-10
Statistics: outcomes	#20a	Statistical methods for analysing primary and secondary outcomes. Reference to where other details of the statistical analysis plan can be found, if not in the protocol	9-12
Statistics: additional analyses	#20b	Methods for any additional analyses (eg, subgroup and adjusted analyses)	9-12

For peer review only

Statistics: analysis population and missing data	#20c	Definition of analysis population relating to protocol non-adherence (eg, as randomised analysis), and any statistical methods to handle missing data (eg, multiple imputation)	9-12
**Methods: Monitoring**			
Data monitoring: formal committee	#21a	Composition of data monitoring committee (DMC); summary of its role and reporting structure; statement of whether it is independent from the sponsor and competing interests; and reference to where further details about its charter can be found, if not in the protocol. Alternatively, an explanation of why a DMC is not needed	N/A - formal commitee not needed as this is a minimal risk trail
Data monitoring: interim analysis	#21b	Description of any interim analyses and stopping guidelines, including who will have access to these interim results and make the final decision to terminate the trial	N/A - as above
Harms	#22	Plans for collecting, assessing, reporting, and managing solicited and spontaneously reported adverse events and other unintended effects of trial interventions or trial conduct	N/A
Auditing	#23	Frequency and procedures for auditing trial conduct, if any, and whether the process will be independent from investigators and the sponsor	N/A - as this is a feasiblity study the process of how the intervention works is part of this.
**Ethics and dissemination**			
Research ethics approval	#24	Plans for seeking research ethics committee / institutional review board (REC / IRB) approval	12
Protocol amendments	#25	Plans for communicating important protocol modifications (eg, changes to eligibility criteria, outcomes, analyses) to relevant parties (eg, investigators, REC / IRBs, trial	12

For peer review only

		participants, trial registries, journals, regulators)	
Consent or assent	#26a	Who will obtain informed consent or assent from potential trial participants or authorised surrogates, and how (see Item 32)	7
Consent or assent: ancillary studies	#26b	Additional consent provisions for collection and use of participant data and biological specimens in ancillary studies, if applicable	7
Confidentiality	#27	How personal information about potential and enrolled participants will be collected, shared, and maintained in order to protect confidentiality before, during, and after the trial	7, 9
Declaration of interests	#28	Financial and other competing interests for principal investigators for the overall trial and each study site	12
Data access	#29	Statement of who will have access to the final trial dataset, and disclosure of contractual agreements that limit such access for investigators	9
Ancillary and post trial care	#30	Provisions, if any, for ancillary and post-trial care, and for compensation to those who suffer harm from trial participation	N/A - participants will continue with their usual HIV care
Dissemination policy: trial results	#31a	Plans for investigators and sponsor to communicate trial results to participants, healthcare professionals, the public, and other relevant groups (eg, via publication, reporting in results databases, or other data sharing arrangements), including any publication restrictions	12
Dissemination policy: authorship	#31b	Authorship eligibility guidelines and any intended use of professional writers	13
Dissemination policy: reproducible research	#31c	Plans, if any, for granting public access to the full protocol, participant-level dataset, and statistical code	12

For peer review only

Appendices

Informed consent materials	#32	Model consent form and other related documentation given to participants and authorised surrogates	Attached as additional document
Biological specimens	#33	Plans for collection, laboratory evaluation, and storage of biological specimens for genetic or molecular analysis in the current trial and for future use in ancillary studies, if applicable	N/A - study does not involve biological specimens

Notes:

- 2b: 1-13 - Listed throughout and in trial registration
- 5c: N/A - sponsors/funders to not have a role in study activities
- 11c: N/A - there is nothing for the participants to adhere to apart from attending the Silver Clinic for their appointment, which they will recive a text reminder about.
- 17b: N/A - there is no change to patients HIV care during the trial, therefore if there was an emergency so they would recieve the same care reagrdless of their trial allocation.
- 18b: N/A - as this is a feasibility trial retention and lost to follow-up are things we want to know about
- 21a: N/A - formal commitee not needed as this is a minimal risk trail
- 21b: N/A - as above
- 23: N/A - as this is a feasiblity study the process of how the intervention works is part of this.
- 30: N/A - participants will continue with their usual HIV care
- 32: Attached as additional document
- 33: N/A - study does not involve biological specimens The SPIRIT Explanation and Elaboration paper is distributed under the terms of the Creative Commons Attribution License CC-BY-NC. This checklist was completed on 21. November 2022 using https://www.goodreports.org/, a tool made by the EQUATOR Network in collaboration with Penelope.ai

For peer review only

BMJ Open

The Silver Clinic: protocol for a feasibility randomised controlled trial of Comprehensive Geriatric Assessment for people living with HIV and frailty

Journal:	*BMJ Open*
Manuscript ID	bmjopen-2022-070590.R1
Article Type:	Protocol
Date Submitted by the Author:	17-Mar-2023
Complete List of Authors:	St. Clair-Sullivan, Natalie; Brighton and Sussex Medical School; King's College London, Cicely Saunders Institute of Palliative Care, Policy & Rehabilitation<br>Bristowe, Katherine; King's College London, Cicely Saunders Institute, Department of Palliative Care, Policy and Rehabilitation<br>Adler, Zoe; University Hospitals Sussex NHS Trust<br>Bremner, Stephen; Brighton and Sussex Medical School<br>Harding, Richard; King's College London, Cicely Saunders Institute, Department of Palliative Care, Policy and Rehabilitation<br>Levett, Thomas; Brighton and Sussex Medical School<br>Maddocks, Matthew; King's College London, Cicely Saunders Institute of Palliative Care, Policy & Rehabilitation<br>Pargeter, Gary; Lunch Positive<br>Roberts, Jonathan; University Hospitals Sussex NHS Trust<br>Yi, Deokhee; King's College London, Department of Palliative Care, Policy and Rehabilitation<br>Vera, Jaime; Brighton and Sussex Medical School
**Primary Subject Heading**:	HIV/AIDS
Secondary Subject Heading:	Evidence based practice, Geriatric medicine, Health services research, Infectious diseases, Patient-centred medicine
Keywords:	HIV & AIDS < INFECTIOUS DISEASES, GERIATRIC MEDICINE, PRIMARY CARE, QUALITATIVE RESEARCH

SCHOLARONE™
Manuscripts

The Silver Clinic: protocol for a feasibility randomised controlled trial of Comprehensive Geriatric Assessment for people living with HIV and frailty

Natalie St Clair-Sullivan[1,2], Katherine Bristowe[2], Zoe Adler[3], Stephen Bremner[1], Richard Harding[2], Thomas Levett[1], Matthew Maddocks[2], Gary Pargeter[4], Jonathan Roberts[3], Deokhee Yi[2], Jaime Vera[1]

[1]Brighton and Sussex Medical School, Brighton, East Sussex, UK; [2]King's College London, Cicely Saunders Institute of Palliative Care, Policy & Rehabilitation, London, UK; [3]University Hospitals Sussex NHS Trust, Brighton, UK; [4]Lunch Positive, Brighton, UK

Correspondence to:

Natalie St Clair-Sullivan

Brighton and Sussex Medical School, Brighton, East Sussex BN1 9PX, UK

N.StClair-Sullivan@bsms.ac.uk

Protocol version 6, 10/08/2022

Word count: 3035

Keywords: HIV, Frail Elderly, Geriatric Assessment, integrated care, trial protocols, feasibility Studies, mixed-methods

For peer review only

ABSTRACT

Introduction

Many people ageing with HIV are also living with multiple comorbidities and geriatric syndromes including frailty and cognitive deterioration. These complex needs can be challenging to meet within existing HIV care services. This study investigates the acceptability and feasibility of screening for frailty and of using a comprehensive geriatric assessment approach, delivered via the Silver Clinic, to support people living with HIV affected by frailty.

Methods and analysis

Mixed-methods, parallel-group, randomised, controlled feasibility trial aiming to recruit 84 people living with HIV ≥50, identified as frail. Participants will be recruited from the HIV unit at the Royal Sussex County Hospital, University Hospitals Sussex NHS Foundation Trust, Brighton, UK.Participants will be randomised 1:1 to receive usual HIV care or the Silver clinic intervention, which uses a Comprehensive Geriatric Assessment approach. Psychosocial, physical and service use outcomes will be measured at baseline, 26 weeks and 52 weeks. Qualitative interviews will be conducted with a subset of participants from both arms. Primary outcome measures include recruitment and retention rates and completion of clinical outcome measures. These will be used in conjunction with a priori progression criteria and the qualitative data (acceptability of trial procedures and intervention) to determine the feasibility and design of a definitive trial.

Ethics and dissemination

This study has been approved by East Midlands – Leicester Central Research Ethics Committee (reference 21/EM/0200). All participants will receive written information about the study and be required to provide informed consent. Results will be disseminated via peer-reviewed journals, conferences, and community engagement.

Trial registration number

ISRCTN14646435.

For peer review only

For peer review only - http://bmjopen.bmj.com/site/about/guidelines.xhtml

Article Summary

Strengths and limitations of this study
• This study will evaluate the feasibility of screening for frailty and applying a comprehensive geriatric assessment, delivered via outpatient HIV services.<br>• The inclusion of qualitative methods will provide an understanding of how to optimise the intervention.<br>• The comprehensive set of outcome measures will capture information about physical and cognitive impairment, overall well-being, social interaction, and healthcare utilisation.<br>• A feasibility randomised controlled trial design allows for testing the acceptability and feasibility of a full-scale trial and refining of the intervention.<br>• It is not possible to blind participants to their trial arm or the healthcare professionals delivering the intervention.

INTRODUCTION

Of the people accessing HIV services in the UK, 39% are now aged 50 and over (1,2). People living with HIV (PLWH) over 50 appear to experience a disproportionate amount of comorbidities in comparison to their HIV negative counterparts, particularly in regard to geriatric syndromes, such as frailty and cognitive deterioration, which they experience at younger ages (3,4). Studies including a younger cohort of PLWH aged 50-64 demonstrate a frailty prevalence comparable to that of HIV negative cohorts aged 65 and older (5). As such, PLWH may not yet have reached the current UK recommended ages for frailty identification advocated in primary, secondary and community care settings (6,7). This coupled with the potential limited access to geriatric and other frailty services based on age alone, runs the risk of delayed identification of frailty, and identification at a more severe stage where interventions may be less effective, resulting in greater health and social care costs (8).

Current models of HIV care are not addressing the needs of people with HIV, with 47% of health care and 62% of social care needs not being met (9). Moreover, current care models may disadvantage older people living with HIV (OPWH) with, or at risk of, frailty as they can bounce between specialist HIV services and primary care. HIV specialist healthcare professionals (HCPs) often lack the awareness and experience to identify and manage frailty, and many GPs lack knowledge and confidence around HIV (10,11). Use of multiple services can be especially challenging for some OPWH who avoid seeking care in non-HIV services because of perceived or experienced stigma and discrimination (12), which is often highlighted in community engagement work (13–15).

To address this problem the British HIV Association (BHIVA) standards of Care for PLWH state that involvement of a geriatrician with HIV knowledge will strengthen service provision, though how to achieve this is unknown (16). The European AIDS Clinical Society (EACS) guidelines recommend

For peer review only - http://bmjopen.bmj.com/site/about/guidelines.xhtml

screening for frailty in people with HIV (17) and whilst tools to identify patients at risk of frailty using scoring methods are increasingly used internationally (18) and have recently been integrated into UK primary care, it is unknown if screening for frailty among OPWH is acceptable, feasible and useful as part of HIV services, particularly for those who are not chronologically elderly. Evidence-based models of care for OPWH at risk of frailty are needed to inform services on how to best to provide care for patients as described by The King's Fund: The future of HIV services in England, shaping the response to changing needs document (19). Two national surveys led by our team (20,21) and work by community organisations (4) underscore the need for evidence-based guidance on how to best to provide care for OPWH.

The Comprehensive Geriatric Assessment (CGA) is a multidimensional, interdisciplinary diagnostic process used to determine the medical, psychosocial, and functional capabilities of older adults. The CGA has been studied both as a hospital-based programme and as an outpatient consultative service (22) (integrated or separate) to other subspecialties of medicine such as haematology (23), nephrology (24), and oncology (25), and in multimorbidity (26) where evidence suggests that screening for frailty and delivering CGA-based care can improve treatment decision making and reduce risk of institutionalisation when applied to other chronic conditions (27,28). Meta-analyses have demonstrated that CGA in older HIV-negative individuals can delay the development of disability, reduce admissions and hospital stays, and improve survival and functional ability (27–29). However, it is not clear whether CGA can improve outcomes for those OPWH with frailty.

There are few geriatric clinics for people with HIV with published data (30–33); most are ageing clinics set up in Europe and the USA, with different objectives according to local circumstances which lack robust evaluation. Therefore, this will be the first study to evaluate the feasibility of screening for frailty and applying the CGA, delivered through a joint HIV-ageing clinic (the 'Silver Clinic') in outpatient HIV services. Our findings can inform the implementation of models of care for PLWH at risk of frailty.

Objectives

The aim of this study is to assess the feasibility and acceptability of screening for frailty in OPWH and the Silver Clinic intervention, using a CGA approach; and to test the feasibility of a randomised controlled trial (RCT) to evaluate this intervention in the wider HIV setting. The main objectives are (1) to determine a sample size and primary outcome for a definitive RCT, and (2) to explore what frailty means, what outcomes matter and the experience of the trial processes for OPWH, including

For peer review only - http://bmjopen.bmj.com/site/about/guidelines.xhtml

communication about the trial, recruitment, randomisation, completion of measures, and experience of participation in the trial. Secondary objectives are to (3) to undertake preliminary cost/service utilization analysis and establish cost analysis outcomes for a definitive trial; (4) evaluate the feasibility and acceptability of implementing frailty screening and the Silver Clinic as part of HIV care; (5) To identify development needs and changes required to optimize the referral pathways, clinic structures and the intervention in preparation for a definitive trial; (6) To explore the acceptability of measures of frailty for OPWH. The objectives of the health economic analysis are: 1) to estimate the costs of the intervention; 2) to understand and estimate the costs of formal health and social care and informal care among patients with HIV and frailty; 3) to examine the feasibility of conducting cost-effectiveness analysis of this intervention in the full trial

METHODS AND ANALYSIS

Trial design

The Silver Clinic feasibility study will use a mixed-method randomised controlled trial design. Participants will be randomised 1:1 to two parallel groups: usual care, or the Silver Clinic intervention, (including the CGA). Quantitative data (including process data and participant outcome measures at baseline, week 26 and week 52) will be collected alongside nested qualitative interview data from a subset of participants.

Setting

Participants will be recruited from the HIV unit at the Royal Sussex County Hospital (RSCH), University Hospitals Sussex NHS Foundation Trust (UHSx), Brighton, UK. The UHSx is an NHS foundation trust consisting of seven hospitals, providing both unscheduled and planned clinical services across Brighton & Hove and West Sussex. Data collection will take place at either the RSCH where participants receive their usual HIV care or at the Clinical Research Facility, RSCH.

Eligibility criteria

Inclusion and exclusion criteria are shown in Table 1.

For peer review only

Table 1. Inclusion and exclusion criteria

Inclusion criteria	Exclusion criteria
PLWH aged 50 years or older with evidence of frailty scoring 3+ on frailty screening, using the FRAIL scale (34)	PLWH aged under 50 or not defined as frail
Consent to contact the GP	Attended the Silver Clinic during the last 12 months

For peer review only

Patient and public involvement

A patient and public representative is a named co-applicant on this study (GP) and is the HIV representative of Community Works for all voluntary HIV organisations. Community Works is the largest network of voluntary organisations in Sussex. They are also the manager of Lunch Positive, a weekly lunch club for people living with HIV providing a community space where OPWH in particular have the opportunity to socialise and access HIV peer support. They will chair the Dissemination Working Group of patient and public involvement and community representatives. Additional PPI representatives recruited during the trial will sit on the dissemination working group. They will be actively involved in the development of the study resources, impact and dissemination strategy and all associated activities. The group will have input on study design and recruitment strategies, review of participant facing materials, input into study conduct, monitoring, evaluation, and dissemination of results to participants, service users, community, and national HIV organisations.

Trial procedures

The schedule of assessments is summarised in Table 2.

For peer review only - http://bmjopen.bmj.com/site/about/guidelines.xhtml

Table 2. Summary of trial procedures

Study visit day	Within 4 weeks Baseline	Day 0	Week 26 +/- 14days	Week 52 (12 months) +/- 14 days
**Description of visit**	**Screening**	**Baseline**	**First follow up**	**Final visit**
Informed consent	X			
Review eligibility	X	X		
Demographic data[1]	X			
Antiretroviral/medical history	X	X	X	X
Healthcare utilisation data[2]		X	X	X
Silver Clinic Consultation (intervention arm only)		X	X	X
Usual care (control arm)		X	X	X
Frailty measures[3]		X	X	X
PRO[4]		X	X	X

[1] Co-morbidities, time since HIV diagnosis, duration living with HIV, CD4, viral load, number of non-ART medications, number of falls in last 6 months.
[2] Number of referrals to primary, secondary and social care.
[3] Fried frailty phenotype measure, FRAIL scale, Timed up and go test, Rockwood clinical frailty scale, Montreal Cognitive Assessment (MoCA).
[4] Patient reported outcomes as HIV PROM, EuroQol, Adult Social Care Outcomes Toolkit (ASCOT), Client Service Receipt inventory (CSRI), Consultation and Relational Empathy (CARE).

Recruitment

84 participants will be recruited from the Royal Sussex County Hospital. Potentially eligible individuals will be identified at their routine HIV annual health check attending the Lawson Unit in Brighton. The HIV annual health check takes place for all service users as part of usual care. The health check is performed by nurses and includes assessment of weight, blood pressure, urinalysis, mental health assessment, sexual health screening, adherence review and cervical cytology and contraception. During this assessment, patients 50 years and over will be screened for frailty using the FRAIL Scale (34). Those with evidence of frailty on their screening will then be informed of the study and if they

express an interest in participating will then be put in contact with the research assistant or nurse to explain the full details of the study, answer any questions, and to give informed consent (see supplemental material). Participants will be consecutively enrolled during the period of recruitment. Recruitment commenced October 2021 and will continue until March 2023, the study is expected to be completed by October 2023.

For those that decline to take part in the study they will be provided with an information leaflet about frailty and their physician will be informed about the frailty screening we have done as part of their

For peer review only - http://bmjopen.bmj.com/site/about/guidelines.xhtml

For peer review only

HIV usual care. Their HIV clinician can refer them to the Silver Clinic as per normal pathways once the feasibility study is complete. These patients will also be asked whether they are happy to share their reasons for declining and if so their answers will be recorded.

Interventions

Study visits

Where possible study visits will be matched up with patient's regular HIV follow up appointments, either at their usual place of HIV care or the Clinical Research Facility, which is located opposite the HIV unit. Silver Clinic visits are offered both in-person and virtually to ensure ease of access to the service, for people living with HIV and frailty.

Usual care

Participants allocated to the control arm will receive healthcare from their HIV physician, GP and community services as standard (see Figure 1). HIV standard of care is provided twice a year and most primary care is provided when actively sought by patients. Participants will be aware of their frailty status, provided with an information leaflet about frailty and will consent to the sharing of the result of their frailty assessment with their GP and HIV physician. Participants will be provided with generic healthy ageing advice but will have no access to the intervention. At the end of 12-months, all control participants, who on assessment continue to require specialist input from ageing experts, will be offered the opportunity to attend the Silver Clinic.

The Silver Clinic

Intervention participants will be reviewed within the Silver Clinic based within the Lawson unit, HIV-service at RSCH. The intervention consists of a CGA approach delivered in a joint HIV geriatrics clinic, providing multidisciplinary assessment and management of geriatric syndromes affecting OPWH including frailty, falls, polypharmacy, multimorbidity, and medication-related problems associated with antiretroviral therapy (ART) (see Figure 1). It also supports OPWH with social and psychological challenges, by formulating health interventions such as physical activity and peer support. The appointment consists of patient history taking, physical examination, blood sample and review of medications, cognition, social and mental health. An individualised care plan will then be generated and sent to the patient's GP/HIV physician. The clinic is delivered once a month with individual appointments of 40 minutes duration for a total of 16 patients per month. Follow-up appointments within the Silver Clinic will be determined by the Silver Clinic physicians and therefore individual to each participant, however it is not expected that it would be more than 2 visits for the duration of the trial. Follow-up frequency in the study will include visits at 6 and 12 months.

For peer review only - http://bmjopen.bmj.com/site/about/guidelines.xhtml

For peer review only

Feasibility outcomes and progression criteria

Primary outcomes: To determine whether a definitive trial is feasible, we will examine the recruitment rates, completion rates of study outcome measures, and retention at specific time points. A priori criteria for trial feasibility and progression to full trial without changes to the trial design are as follows (see Table 3 For further details):

- Recruitment of 60% of eligible patients;
- Recruitment of 84 patients within 6 months; from first patient randomised;
- Retention of 70 participants (allowing up to 15% attrition) to primary end point (6 months);
- Outcome measure completion for 90% of available participants at each time point.

Secondary outcomes: Health service utilisation (CSRI), social care (ASCOT) and physical and mental health components of the HIV PROM and EuroQol index score and visual analogue scale at 6 and 12 months. In addition, satisfaction with care will also be measured at each timepoint using the CARE measure.

Table 3. Silver study feasibility outcomes, contributing data and progression criteria

Objective	Feasibility outcomes	Contributing data	Progression criteria Green	Amber	Red

For peer review only - http://bmjopen.bmj.com/site/about/guidelines.xhtml

1	*Identification & recruitment of eligible patients	Screening & recruitment log	≥60% eligible recruited	59-40%	<40%
3	*Retention of participants at follow up	Participation data	≥70 pts at 6 months<br>≥55 pts at 12 months	74-60%<br>59-40%	<60%<br><40%
4	Contamination of the control arm	CGA service data	≥10% participants receive a CGA within usual care	≥11-20%	≥20%
5	*Outcome measure completion	Participation data	Missing data of ≤10% for each measure.<br>Participant-reported acceptability.	11-25%<br>Some	>25%<br>None
6	Participant satisfaction with care	Participant questionnaires & interviews	Reported as acceptable (or can be with minimal modification)	Reported as acceptable with modification	Intervention not acceptable

*Primary focus; Traffic-light progression criteria(35,36) - Green: likely no concerning issues, Amber: potentially remediable issues , Red: potentially intractable issues.

Data collection

Baseline demographic data will be collected including personal characteristics (age, gender, sex at birth, ethnicity) and social factors (marital status, employment status, residential status, formal education level, annual income) and comorbidities. Demographic data and patient record data capture for the enrolment and follow-up forms will be done by manual data keying or electronically. Manual data keying is performed in a secure online browser-based platform called REDCap. Electronic data capture entails local extraction of data from clinical electronic databases and will be stored securely on the UHSx systems, HIV drive. Only the research team (including research administrator) will have access to this data and will not be made available outside the team or institution.

Process data will be collected to understand intervention delivery and trial design appropriateness. For the Silver Clinic intervention, records pertaining to CGA date, recommendations and follow up will be collected. For trial process data, trial screening, recruitment rates, participation at each timepoint and amount of missing data will be recorded.

Standardised clinical outcome measures that represent multiple health and healthcare service domains will be collected at baseline, week 26 and week 52 post randomisation. The Positive

For peer review only - http://bmjopen.bmj.com/site/about/guidelines.xhtml

Outcomes HIV PROM measures multidimensional symptoms and concerns for PLWH (37,38); the EuroQol EQ-5D-5L measures health related quality of life (39); the Adult Social Care Outcomes Toolkit (ASCOT) measures Social care related quality of life (40); the Client Service Receipt Inventory (CSRI) measures services and support accessed (41); the Consultation and relational empathy measure (CARE) is used to assess interpersonal quality of healthcare encounters (42); the Fried Frailty Phenotype and FRAIL Scale are used to assess physical frailty (34,43); and the Timed Up and Go test to assess functional mobility and falls risk (44). Physical tests will be conducted by the researcher and questionnaires will be completed with support of the researcher.

Nested qualitative interviews

OPWH will be recruited for qualitative interview via purposive sampling from within the RCT participants. OPWH will be purposively sampled by trial arm, age, gender, duration of HIV diagnosis, ethnicity, sexual orientation, living situation and frailty score, to ensure a maximum variation sample. Members of the study team will meet regularly to discuss ongoing recruitment and the characteristics of the recruited sample. This will allow for the identification of characteristics not yet included in the study and to purposively target these in subsequent participants, ensuring diversity in the overall sample.

A purposive sample of up to 15 participants from each arm of the trial will be interviewed on completion of trial participation to examine experiences of: recruitment to the trial, management of their priority concerns during the course of the trial, referral to the Silver Clinic, the description of CGA, experience of the Silver Clinic and perceived impact upon priority outcomes (intervention arm only), satisfaction with care and acceptability of participating in an RCT of the Silver Clinic intervention for OPWH. Draft topic guides will be reviewed by PPI members. Face-to-face, telephone or video call interviews will be conducted by the research assistant and take place in a location of the participant's choosing. Interviews will be digitally audio recorded. Field notes will be used to record contextual factors, participant responses, and personal reflections.

Sample size

This is a feasibility trial and therefore not powered to test effectiveness of the intervention compared to standard treatment. However, data will be used to inform the sample size calculation for a future definitive trial. To precisely estimate the standard deviation of the primary outcome for a future trial, a sample size of at least 35 participants per arm is recommended(45,46). Based on the local patient numbers (cohort of 2450; 54% over 50 years old), we anticipate recruiting 42 patients per arm i.e. 84 in all allowing for attrition of 15% to be achievable.

For peer review only - http://bmjopen.bmj.com/site/about/guidelines.xhtml

For peer review only

Randomisation and blinding

After the baseline assessment the research assistant will randomise participants using REDCap in a 1:1 allocation to receive either usual care (Control arm) or referral to the Silver Clinic (Intervention arm), stratifying one age (50-56, 66-80, 81-95, 96-110) and sex to ensure a balanced sample in both arms. Clinicians delivering the intervention will be blinded to the screening and randomisation process as they will have no knowledge of when patients are screened for frailty nor have any influence on the randomisation process, minimising the possibility of selection bias.

Analysis

Quantitative data

Baseline characteristics of the intervention and control participants will be summarised using descriptive statistics. Participant flow through the trial will be shown on a flowchart according to the CONSORT 2010 Statement extension for pilot and feasibility trials (47). Data will be presented by trial arm. Normally distributed variables will be summarised by their means and standard deviations, skewed continuous variables by their medians and interquartile ranges and categorical variables by their frequencies and percentages. For the feasibility outcomes, proportion of patients recruited, participants retained and data completeness 95% confidence intervals will be presented. Differences in means between arms for the secondary outcomes will be presented with 95% confidence intervals. Analysis will be of available cases following intention to treat principles. Missing data will be quantified but not imputed. A full statistical analysis plan will be agreed prior to database lock for the final analysis.

Qualitative data

Interview and focus group recordings will be transcribed verbatim and pseudonymised (removing any identifiable characteristics). Interviews will be analysed using reflexive thematic analysis, in six stages: familiarisation; coding; searching; reviewing; defining themes; and reporting (48,49). Analysis will be reviewed by the study team, including PPI members, and revisited to develop a theoretical model of person-centred care for OPWH and frailty. Analysis will be supported using NVivo qualitative data analysis software, and reported in accordance with the consolidated criteria for reporting qualitative studies (COREQ) (50). These results will be reviewed alongside our previous qualitative study (51)exploring the perspectives of PLWH and their healthcare professionals on frailty and frailty screening, to understand how HIV provider experiences and perspectives may contribute to the provision of frailty services and inform the subsequent refined intervention.

Cost analysis

For peer review only - http://bmjopen.bmj.com/site/about/guidelines.xhtml

Data for costs will be collected by the modified Client Service Receipt Inventory (CSRI), which asks patients about the health and social care service use and informal care provided by family and/or friends. Response rate for EQ-5D-5L and visual analog scale at different time points will be checked and described. EQ-5D index scores will be calculated using the Crosswalk value set using EQ-5D-3L in the UK as recommended by the NICE(52). We will examine the completion rates for each item in outcome measurements and CSRI first. Unit costs for each service item will be collected from usual data sources (e.g. NHS Reference costs, PSSRU Unit costs of health and social care, market wage rates). Then, we will describe and compare the utilization and costs of formal care (health and social care) and informal care provided by family/friends. Costs of caring for patients in this group are of interest to commissioning purposes. We will describe the patterns of service uses and costs by types of services (e.g. acute care, community care) at different time points. The intervention costs will be estimated using records from trial management teams and CSRI. We will try calculating incremental cost-effectiveness ratios (ICERs) of this intervention although we do not aim to use the results to justify the cost-effectiveness of the intervention. We will also explore the uncertainties around the parameters and draw the cost-effectiveness planes using bootstrapping. Because this is the feasibility RCT, we will not be able to make a conclusive remark on cost-effectiveness of the intervention.

Health economic analysis from an NHS perspective uses formal care costs but formal and informal care costs will be used for analysis from a wider societal perspective. We have also included questions about the changes in labour market activities (e.g. stopping working or reducing hours of working due to illness) to investigate the feasibility of including social costs in future economic analysis.

ETHICS AND DISSEMINATION

This study has been approved by East Midlands – Leicester Central Research Ethics Committee (REC reference: 21/EM/0200). Protocol amendments will be communicated to all relevant parties. Prior to the study start patients will be informed verbally by their HIV doctor or study nurse about the study and will receive written information about the study. Informed consent will be obtained before any study activities can begin (see supplemental material). The findings from this study, positive, negative or inconclusive, are intended to be published in peer-reviewed journals and/or presented at conferences and seminars and disseminated through HIV community groups.

Competing interests

None declared.

For peer review only - http://bmjopen.bmj.com/site/about/guidelines.xhtml

For peer review only

Funding

This project is funded by the National Institute for Health and Care Research (NIHR) under its Research for Patient Benefit (RfPB) Programme (Grant Reference Number NIHR201060). The views expressed are those of the author(s) and not necessarily those of the NIHR or the Department of Health and Social Care. MM is funded by a National Institute for Health and Care Research (NIHR) Career Development Fellowship (CDF-2017–10-009) and the NIHR Applied Research Collaboration South London (NIHR ARC South London) at King's College Hospital NHS Foundation Trust.

Contributors

KB and JV led on the design of the study. NS drafted the protocol and all authors (NS, KB, ZA, SB, RH, TL, MM, GP, JR, DY, JV) contributed to the study design, writing, planning and revising the protocol, including the safety assessments. SB wrote the statistical section and analysis plan and DY wrote the cost analysis section. GP, PPI member, helped develop trial related materials such as the PIS and interview topic guides and contributed to the dissemination strategy. All authors (NS, KB, ZA, SB, RH, TL, MM, GP, JR, DY, JV) contributed to and approved the final published version of the trial protocol.

Trial sponsor

Trial Sponsor: University of Sussex

Sponsor's Reference: 087 VER/300599

Contact name: Anthony Walsh

Address: Senior Research Ethics & Integrity Officer / Research and Enterprise Services 1st Floor, Falmer House, University of Sussex, Brighton BN1 9QF, United Kingdom

Telephone: +44 (0)1273 872748

Email: researchsponsorship@sussex.ac.uk

References

1. Levett T, Alford K, Roberts J, Adler Z, Wright J, Vera JH. Evaluation of a combined HIV and geriatrics clinic for older people living with HIV: the Silver clinic in Brighton, UK. Geriatrics [Internet]. 2020 [cited 2020 Dec 1];5(81). Available from: http://sro.sussex.ac.uk

2. Piot P, Abdool Karim SS, Hecht R, Legido-Quigley H, Buse K, Stover J, et al. Defeating AIDS - Advancing global health. Lancet. 2015;386(9989):171–218.

3. Bloch M. Frailty in people living with HIV. AIDS Res Ther [Internet]. 2018 Dec 16 [cited 2020 Mar 30];15(1):19. Available from: https://aidsrestherapy.biomedcentral.com/articles/10.1186/s12981-018-0210-2

4. Terrence Higgins Trust. Uncharted Territory A report into the first generation growing older with HIV. 2017.

For peer review only - http://bmjopen.bmj.com/site/about/guidelines.xhtml

5. Levett TJ, Cresswell F V., Malik MA, Fisher M, Wright J. Systematic Review of Prevalence and Predictors of Frailty in Individuals with Human Immunodeficiency Virus. J Am Geriatr Soc [Internet]. 2016 May 1 [cited 2021 May 11];64(5):1006–14. Available from: https://pubmed.ncbi.nlm.nih.gov/27225356/

6. Vernon MJ. NHS RightCare: Frailty Toolkit. 2019.

7. British Geriatrics Society. Fit for Frailty Part 1 [Internet]. 2017 [cited 2021 May 14]. Available from: https://www.bgs.org.uk/sites/default/files/content/resources/files/2018-05-23/fff_full.pdf

8. Lundqvist M, Alwin J, Henriksson M, Husberg M, Carlsson P, Ekdahl AW. Cost-effectiveness of comprehensive geriatric assessment at an ambulatory geriatric unit based on the AGe-FIT trial. BMC Geriatr [Internet]. 2018 Jan 31 [cited 2021 May 14];18(1). Available from: https://pubmed.ncbi.nlm.nih.gov/29386007/

9. Public Health England. Positive Voices: The National Survey of People Living with HIV Findings from the 2017 survey [Internet]. 2017 [cited 2021 May 11]. Available from: moz-extension://eacf2752-a225-4ca0-8bae-4f106db5688e/enhanced-reader.html?openApp&pdf=https%3A%2F%2Fassets.publishing.service.gov.uk%2Fgovernment%2Fuploads%2Fsystem%2Fuploads%2Fattachment_data%2Ffile%2F857922%2FPHE_positive_voices_report_2019.pdf

10. Liddy C, Shoemaker ES, Crowe L, Boucher LM, Rourke SB, Rosenes R, et al. How the delivery of HIV care in Canada aligns with the Chronic Care Model: A qualitative study. PLoS One [Internet]. 2019 Jun 1 [cited 2021 Aug 25];14(7):e0220516. Available from: https://journals.plos.org/plosone/article?id=10.1371/journal.pone.0220516

11. Siegler EL, Burchett CO, Glesby MJ. Older people with HIV are an essential part of the continuum of HIV care. J Int AIDS Soc [Internet]. 2018;21:25188. Available from: http://onlinelibrary.wiley.com/doi/10.1002/jia2.25188/full%7Chttps://doi.org/10.1002/jia2.25188

12. StigmaSurveyUK. HIV in the UK: Changes and Challenges; Actions and Answers The People Living With HIV Stigma Survey UK 2015 Women [Internet]. 2015 [cited 2020 Oct 20]. Available from: http://www.nat.org.uk/media/Files/PDF documents/Mori_2014_report_FINAL.pdf

13. Nixon E, Cooper V, Youssef E, Smith H, Robert G, Fisher M. NH4 Service development for people with HIV who have comorbid conditions using experience-based co-design methodology. Sex Transm Infect [Internet]. 2016 Jun 1 [cited 2021 May 14];92(Suppl 1):A18.1-A18. Available from: https://sti.bmj.com/content/92/Suppl_1/A18.1

14. Pollard A, Llewellyn C, Cooper V, Sachikonye M, Perry N, Nixon E, et al. Patients' perspectives on the development of HIV services to accommodate ageing with HIV: a qualitative study. Int J STD AIDS [Internet]. 2018 Apr 1 [cited 2021 May 14];29(5):483–90. Available from: https://journals.sagepub.com/doi/abs/10.1177/0956462417735723

15. Miners AH, Llewellyn CD, Cooper VL, Youssef E, Pollard AJ, Lagarde M, et al. A discrete choice experiment to assess people living with HIV's (PLWHIV's) preferences for GP or HIV clinic appointments. Sex Transm Infect [Internet]. 2017 Mar 1 [cited 2021 May 14];93(2):105–11. Available from: http://sti.bmj.com/

16. BHIVA. Standards of Care for People Living with HIV [Internet]. 2018. Available from: https://www.bhiva.org/file/KrfaFqLZRlBhg/BHIVA-Standards-of-Care-2018.pdf

17. European AIDS Clinical Society. European AIDS Clinical Society Guidelines [Internet]. 2019.

For peer review only - http://bmjopen.bmj.com/site/about/guidelines.xhtml

Available from: https://wwweacsocietyorg/files/2019_guidelines-100_finalpdf

18. Romero-Ortuno R, Soraghan C. A Frailty Instrument for primary care for those aged 75 years or more: Findings from the Survey of Health, Ageing and Retirement in Europe, a longitudinal population-based cohort study (SHARE-FI75+). BMJ Open [Internet]. 2014 Dec 1 [cited 2021 May 11];4(12):6645. Available from: http://bmjopen.bmj.com/

19. Baylis A, Buck D, Anderson J, Jabbal J, Ross S. The future of HIV services in England Shaping the response to changing needs [Internet]. 2017 [cited 2019 Aug 16]. Available from: https://www.kingsfund.org.uk/sites/default/files/field/field_publication_file/Future_HIV_services_England_Kings_Fund_April_2017.pdf

20. Cresswell F, Levett T. Specialist care of older adults with HIV infection in the UK: a service evaluation. HIV Med [Internet]. 2017 Aug 1 [cited 2021 Feb 9];18(7):519–24. Available from: http://doi.wiley.com/10.1111/hiv.12481

21. Youssef E, Wright J, Cooper V, Nixon E, Vera J. Current models of care for the management of HIV patients with comorbidities in England: a survey. J Int AIDS Soc. 2016;19(7).

22. Smith SM, Wallace E, O'Dowd T, Fortin M. Interventions for improving outcomes in patients with multimorbidity in primary care and community settings [Internet]. Vol. 2016, Cochrane Database of Systematic Reviews. John Wiley and Sons Ltd; 2016 [cited 2021 May 11]. Available from: https://pubmed.ncbi.nlm.nih.gov/26976529/

23. Hamaker ME, Mitrovic M, Stauder R. The G8 screening tool detects relevant geriatric impairments and predicts survival in elderly patients with a haematological malignancy. Ann Hematol [Internet]. 2014 [cited 2021 May 11];93(6):1031–40. Available from: https://pubmed.ncbi.nlm.nih.gov/24488257/

24. Kallenberg MH, Kleinveld HA, Dekker FW, van Munster BC, Rabelink TJ, van Buren M, et al. Functional and cognitive impairment, frailty, and adverse health outcomes in older patients reaching ESRD-a systematic review. Clin J Am Soc Nephrol [Internet]. 2016 [cited 2021 May 11];11(9):1624–39. Available from: https://pubmed.ncbi.nlm.nih.gov/27342598/

25. Caillet P, Laurent M, Bastuji-Garin S, Liuu E, Culine S, Lagrange JL, et al. Optimal management of elderly cancer patients: Usefulness of the comprehensive geriatric assessment. Clin Interv Aging [Internet]. 2014 Sep 29 [cited 2021 May 11];9:1645–60. Available from: /pmc/articles/PMC4189720/

26. van Loon IN, Wouters TR, Boereboom FTJ, Bots ML, Verhaar MC, Hamaker ME. The relevance of geriatric impairments in patients starting dialysis: A systematic review. Clin J Am Soc Nephrol [Internet]. 2016 Apr 26 [cited 2021 May 11];11(7):1245–59. Available from: https://europepmc.org/articles/PMC4934838

27. Ellis G, Gardner M, Tsiachristas A, Langhorne P, Burke O, Harwood RH, et al. Comprehensive geriatric assessment for older adults admitted to hospital. Cochrane Database Syst Rev [Internet]. 2017 Sep 12 [cited 2021 May 11];(9):1–108. Available from: https://pubmed.ncbi.nlm.nih.gov/28898390/

28. Garrard JW, Cox NJ, Dodds RM, Roberts HC, Sayer AA. Comprehensive geriatric assessment in primary care: a systematic review [Internet]. Vol. 32, Aging Clinical and Experimental Research. Springer; 2020 [cited 2021 Feb 15]. p. 197–205. Available from: https://doi.org/10.1007/s40520-019-01183-w

29. Stuck AE, Iliffe S. Comprehensive geriatric assessment for older adults [Internet]. Vol. 343, BMJ (Online). BMJ; 2011 [cited 2021 May 11]. p. 1029–30. Available from:

For peer review only - http://bmjopen.bmj.com/site/about/guidelines.xhtml

https://pubmed.ncbi.nlm.nih.gov/22034147/

30. Siegler EL, Burchett CO, Glesby MJ. Older people with HIV are an essential part of the continuum of HIV care [Internet]. Vol. 21, Journal of the International AIDS Society. John Wiley and Sons Inc.; 2018 [cited 2021 May 11]. p. 25188. Available from: /pmc/articles/PMC6178281/

31. Ruiz M, Cefalu C, Ogbuokiri J. A dedicated screening program for geriatric HIV-infected patients integrating HIV and geriatric care. J Int Assoc Physicians AIDS Care [Internet]. 2010 May [cited 2021 May 11];9(3):157–61. Available from: https://pubmed.ncbi.nlm.nih.gov/20530469/

32. Ruiz M, Cefalu C. Characteristics of frail patients in a geriatric-HIV program: The experience of an urban academic center at one year follow-up. J Int Assoc Physicians AIDS Care [Internet]. 2011 May 18 [cited 2021 May 11];10(3):138–43. Available from: http://jiapac.sagepub.com

33. Waters L, Patterson B, Scourfield A, Hughes A, de Silva S, Gazzard B, et al. A dedicated clinic for HIV-positive individuals over 50 years of age: A multidisciplinary experience [Internet]. Vol. 23, International Journal of STD and AIDS. SAGE PublicationsSage UK: London, England; 2012 [cited 2021 May 11]. p. 546–52. Available from: https://journals.sagepub.com/doi/abs/10.1258/ijsa.2012.011412

34. Abellan Van Kan G, Rolland Y, Bergman H, Morley JE, Kritchevsky SB, Vellas B. The I.A.N.A. task force on frailty assessment of older people in clinical practice. J Nutr Heal Aging [Internet]. 2008 Jan [cited 2021 May 14];12(1):29–37. Available from: https://pubmed.ncbi.nlm.nih.gov/18165842/

35. Craig P, Dieppe P, Macintyre S, Michie S, Nazareth I, Petticrew M. Developing and evaluating complex interventions: The new Medical Research Council guidance. Int J Nurs Stud. 2013;50(5):587–92.

36. Brighton LJ, Evans CJ, Farquhar M, Bristowe K, Kata A, Higman J, et al. Integrating Comprehensive Geriatric Assessment for people with COPD and frailty starting pulmonary rehabilitation: the Breathe Plus feasibility trial protocol. ERJ Open Res [Internet]. 2021 Feb 4 [cited 2021 Mar 5];00717–2020. Available from: https://doi.org/10.1183/23120541.00717-2020

37. Bristowe K, Clift P, James R, Josh J, Platt M, Whetham J, et al. Towards person-centred care for people living with HIV : what core outcomes matter, and how might we assess them? A cross-national multi-centre qualitative study with key stakeholders. HIV Med [Internet]. 2019 Jun 4 [cited 2020 Mar 30];20(8):hiv.12758. Available from: https://onlinelibrary.wiley.com/doi/abs/10.1111/hiv.12758

38. Bristowe K, Murtagh FEM, Clift P, James R, Josh J, Platt M, et al. The development and cognitive testing of the positive outcomes HIV PROM: A brief novel patient-reported outcome measure for adults living with HIV. Health Qual Life Outcomes. 2020;18(1):1–10.

39. Rabin R, De Charro F. EQ-5D: A measure of health status from the EuroQol Group. In: Annals of Medicine [Internet]. Royal Society of Medicine Press Ltd; 2001 [cited 2021 May 14]. p. 337–43. Available from: https://pubmed.ncbi.nlm.nih.gov/11491192/

40. Netten A, Burge P, Malley J, Potoglou D, Towers AM, Brazier J, et al. Outcomes of social care for adults: Developing a preference-weighted measure. Health Technol Assess (Rockv) [Internet]. 2012 [cited 2021 May 14];16(16):1–165. Available from: https://pubmed.ncbi.nlm.nih.gov/22459668/

For peer review only - http://bmjopen.bmj.com/site/about/guidelines.xhtml

41. Beecham J, Snell T, Perkins M, Knapp M. Health and social care costs for young adults with epilepsy in the UK. Heal Soc Care Community [Internet]. 2010 Sep [cited 2021 May 14];18(5):465–73. Available from: https://pubmed.ncbi.nlm.nih.gov/20491967/

42. Mercer SW, Maxwell M, Heaney D, Watt GCM. The consultation and relational empathy (CARE) measure: Development and preliminary validation and reliability of an empathy-based consultation process measure. Fam Pract [Internet]. 2004 Dec [cited 2021 May 14];21(6):699–705. Available from: https://pubmed.ncbi.nlm.nih.gov/15528286/

43. Fried LP, Tangen CM, Walston J, Newman AB, Hirsch C, Gottdiener J, et al. Frailty in older adults: Evidence for a phenotype. Journals Gerontol - Ser A Biol Sci Med Sci [Internet]. 2001 [cited 2021 May 11];56(3). Available from: https://pubmed.ncbi.nlm.nih.gov/11253156/

44. Richardson S. The Timed “Up & Go”: A Test of Basic Functional Mobility for Frail Elderly Persons. J Am Geriatr Soc [Internet]. 1991 [cited 2021 May 14];39(2):142–8. Available from: https://pubmed.ncbi.nlm.nih.gov/1991946/

45. Sim J, Lewis M. The size of a pilot study for a clinical trial should be calculated in relation to considerations of precision and efficiency. J Clin Epidemiol [Internet]. 2012 Mar 1 [cited 2021 May 14];65(3):301–8. Available from: http://www.jclinepi.com/article/S0895435611002642/fulltext

46. Teare MD, Dimairo M, Shephard N, Hayman A, Whitehead A, Walters SJ. Sample size requirements to estimate key design parameters from external pilot randomised controlled trials: A simulation study. Trials [Internet]. 2014 Jul 3 [cited 2021 May 14];15(1):1–13. Available from: http://www.trialsjournal.com/content/15/1/264

47. Eldridge SM, Chan CL, Campbell MJ, Bond CM, Hopewell S, Thabane L, et al. CONSORT 2010 statement: Extension to randomised pilot and feasibility trials. BMJ [Internet]. 2016 [cited 2021 May 25];355. Available from: https://pubmed.ncbi.nlm.nih.gov/27777223/

48. Braun V, Clarke V. Using thematic analysis in psychology. Qual Res Psychol [Internet]. 2006 [cited 2018 Jul 25];3(2):77–101. Available from: http://www.tandfonline.com/action/journalInformation?journalCode=uqrp20

49. Braun V, Clarke V. Reflecting on reflexive thematic analysis. Qual Res Sport Exerc Heal [Internet]. 2019 Aug 8 [cited 2020 Feb 28];11(4):589–97. Available from: https://www.tandfonline.com/doi/full/10.1080/2159676X.2019.1628806

50. Tong A, Sainsbury P, Craig J. Consolidated criteria for reporting qualitative research (COREQ): A 32-item checklist for interviews and focus groups. Int J Qual Heal Care. 2007;19(6):349–57.

51. St Clair-Sullivan N, Simmons K, Harding-Swale R, Levett T, Maddocks M, Roberts J, et al. Frailty and frailty screening: A qualitative study to elicit perspectives of people living with HIV and their healthcare professionals. HIV Med. 2022;(June):1–11.

52. National Institute for Health and Care Excellence. Position statement on use of the EQ-5D-5L value set for England (updated October 2019) | Technology appraisal guidance | NICE guidance | Our programmes | What we do | About | NICE [Internet]. NICE; 2019 [cited 2022 Nov 18]. Available from: https://www.nice.org.uk/about/what-we-do/our-programmes/nice-guidance/technology-appraisal-guidance/eq-5d-5l

FIGURE TITLE

For peer review only - http://bmjopen.bmj.com/site/about/guidelines.xhtml

Figure 1. Usual HIV care vs the Silver Clinic intervention

For peer review only

For peer review only - http://bmjopen.bmj.com/site/about/guidelines.xhtml

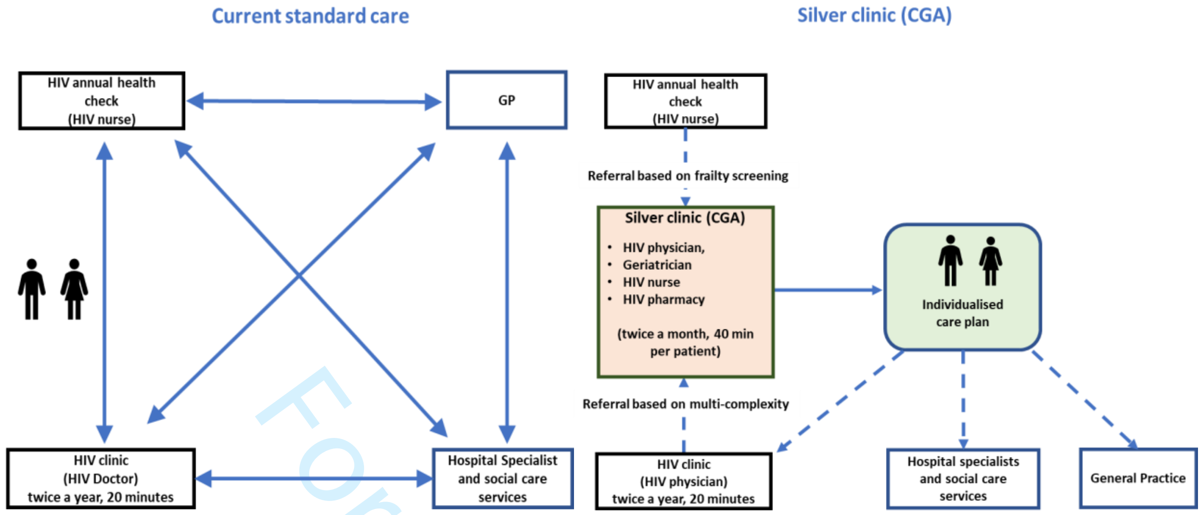

For peer review only

University Hospitals Sussex
NHS Foundation Trust

UNIVERSITY OF SUSSEX

CONSENT FORM FOR PROJECT PARTICIPANTS

Title of Project: Feasibility and acceptability of case-finding and subsequent comprehensive geriatric assessment intervention for older people with HIV comprehensive geriatric assessment intervention for older people with HIV

Name of Principal Investigator: Dr Jaime Vera

Health Research Authority Ethics Committee. Ref No: 21/EM/0200

Please state yes or no:

1 I confirm that I have read and understood the information sheet dated 15/11/2021 (version 4). I have had the chance to ask questions about the study and am satisfied with the answers I have been given.

2 I understand that my participation is voluntary and that I am free to withdraw at any time without giving any reason, without my medical care or legal rights being affected.

3 I understand that relevant sections of my medical notes and data collected during the study may be looked at by researchers from Brighton & Sussex Medical School, from regulatory authorities or from the NHS Trust, where it is relevant to my taking part in this research. I give permission for these individuals to have access to my records.

4 I am happy to be contacted about being involved in a one-to-one interview during the study period (optional).

5 If I choose to take part in the interview, I consent to being interviewed by the researcher (only applicable if YES to No 4).

6 I agree to allowing the interview to be audio recorded and the possible use of quotes, that have been anonymised so that I cannot be identified, to be used in any written study reports. (only applicable if YES to No 4).

7 I consent to the processing of my personal information and data for the purposes of this research study. I understand that such information will be treated as confidential and handled in accordance with data protection legislation.

8 I agree to my GP being informed of my participation in the study.

9 I agree to my medical records being accessed for the purposes of this research study.

Consent form- IRAS Project ID: **300599**
V3 – The silver clinic study
14/09/2021

For peer review only

UNIVERSITY OF SUSSEX

University Hospitals Sussex
NHS Foundation Trust

10 I understand that the information held and maintained by University Hospitals Sussex may be used to help contact me or provide information about my health status.

11 I wish to receive a summary of the study results.

12 I would like to receive a summary of the findings of the study when they have been published by email or by post (please insert either email or postal address below).

Email:

Address:

13 I consent to take part in the above study.

For peer review only

Name of Participant	Date	Signature

Name of Researcher or Person Seeking Consent (If different from researcher)	Date	Signature

When completed: 1 copy for the participant; 1 copy for the researcher site file; *1 copy (original) to be kept in medical notes*

Consent form- IRAS Project ID: **300599**
V3 – The silver clinic study
14/09/2021

Reporting checklist for protocol of a clinical trial.

Based on the SPIRIT guidelines.

Instructions to authors

Complete this checklist by entering the page numbers from your manuscript where readers will find each of the items listed below.

Your article may not currently address all the items on the checklist. Please modify your text to include the missing information. If you are certain that an item does not apply, please write "n/a" and provide a short explanation.

Upload your completed checklist as an extra file when you submit to a journal.

In your methods section, say that you used the SPIRITreporting guidelines, and cite them as:

Chan A-W, Tetzlaff JM, Gøtzsche PC, Altman DG, Mann H, Berlin J, Dickersin K, Hróbjartsson A, Schulz KF, Parulekar WR, Krleža-Jerić K, Laupacis A, Moher D. SPIRIT 2013 Explanation and Elaboration: Guidance for protocols of clinical trials. BMJ. 2013;346:e7586

		Reporting Item	Page Number
**Administrative information**			
Title	#1	Descriptive title identifying the study design, population, interventions, and, if applicable, trial acronym	1
Trial registration	#2a	Trial identifier and registry name. If not yet registered, name of intended registry	1
Trial registration: data set	#2b	All items from the World Health Organization Trial Registration Data Set	1-13 - Listed throughout and in trial registration
Protocol version	#3	Date and version identifier	1
Funding	#4	Sources and types of financial, material, and other support	12
Roles and responsibilities: contributorship	#5a	Names, affiliations, and roles of protocol contributors	13

For peer review only

Roles and responsibilities: sponsor contact information	#5b	Name and contact information for the trial sponsor	13
Roles and responsibilities: sponsor and funder	#5c	Role of study sponsor and funders, if any, in study design; collection, management, analysis, and interpretation of data; writing of the report; and the decision to submit the report for publication, including whether they will have ultimate authority over any of these activities	N/A - sponsors/funders to not have a role in study activities
Roles and responsibilities: committees	#5d	Composition, roles, and responsibilities of the coordinating centre, steering committee, endpoint adjudication committee, data management team, and other individuals or groups overseeing the trial, if applicable (see Item 21a for data monitoring committee)	6, 9-11
**Introduction**			
Background and rationale	#6a	Description of research question and justification for undertaking the trial, including summary of relevant studies (published and unpublished) examining benefits and harms for each intervention	3-4
Background and rationale: choice of comparators	#6b	Explanation for choice of comparators	3-5
Objectives	#7	Specific objectives or hypotheses	4-5
Trial design	#8	Description of trial design including type of trial (eg, parallel group, crossover, factorial, single group), allocation ratio, and framework (eg, superiority, equivalence, non-inferiority, exploratory)	5

Methods:

For peer review only

**Participants, interventions, and outcomes**			
Study setting	#9	Description of study settings (eg, community clinic, academic hospital) and list of countries where data will be collected. Reference to where list of study sites can be obtained	5
Eligibility criteria	#10	Inclusion and exclusion criteria for participants. If applicable, eligibility criteria for study centres and individuals who will perform the interventions (eg, surgeons, psychotherapists)	5, 7-8
Interventions: description	#11a	Interventions for each group with sufficient detail to allow replication, including how and when they will be administered	7-8
Interventions: modifications	#11b	Criteria for discontinuing or modifying allocated interventions for a given trial participant (eg, drug dose change in response to harms, participant request, or improving / worsening disease)	7
Interventions: adherance	#11c	Strategies to improve adherence to intervention protocols, and any procedures for monitoring adherence (eg, drug tablet return; laboratory tests)	N/A - there is nothing for the participants to adhere to apart from attending the Silver Clinic for their appointment, which they will recive a text reminder about.
Interventions: concomitant care	#11d	Relevant concomitant care and interventions that are permitted or prohibited during the trial	7
Outcomes	#12	Primary, secondary, and other outcomes, including the specific measurement variable (eg, systolic blood pressure), analysis metric (eg, change from baseline, final value, time	8-9

For peer review only

		to event), method of aggregation (eg, median, proportion), and time point for each outcome. Explanation of the clinical relevance of chosen efficacy and harm outcomes is strongly recommended	
Participant timeline	#13	Time schedule of enrolment, interventions (including any run-ins and washouts), assessments, and visits for participants. A schematic diagram is highly recommended (see Figure)	6
Sample size	#14	Estimated number of participants needed to achieve study objectives and how it was determined, including clinical and statistical assumptions supporting any sample size calculations	10
Recruitment	#15	Strategies for achieving adequate participant enrolment to reach target sample size	6-7
**Methods: Assignment of interventions (for controlled trials)**			
Allocation: sequence generation	#16a	Method of generating the allocation sequence (eg, computer-generated random numbers), and list of any factors for stratification. To reduce predictability of a random sequence, details of any planned restriction (eg, blocking) should be provided in a separate document that is unavailable to those who enrol participants or assign interventions	11
Allocation concealment mechanism	#16b	Mechanism of implementing the allocation sequence (eg, central telephone; sequentially numbered, opaque, sealed envelopes), describing	11

For peer review only

		any steps to conceal the sequence until interventions are assigned	
Allocation: implementation	#16c	Who will generate the allocation sequence, who will enrol participants, and who will assign participants to interventions	11
Blinding (masking)	#17a	Who will be blinded after assignment to interventions (eg, trial participants, care providers, outcome assessors, data analysts), and how	11
Blinding (masking): emergency unblinding	#17b	If blinded, circumstances under which unblinding is permissible, and procedure for revealing a participant's allocated intervention during the trial	N/A - there is no change to patients HIV care during the trial, therefore if there was an emergency so they would recieve the same care reagrdless of their trial allocation.
**Methods: Data collection, management, and analysis**			
Data collection plan	#18a	Plans for assessment and collection of outcome, baseline, and other trial data, including any related processes to promote data quality (eg, duplicate measurements, training of assessors) and a description of study instruments (eg, questionnaires, laboratory tests) along with their reliability and validity, if known. Reference to where data collection forms can be found, if not in the protocol	9-10
Data collection plan: retention	#18b	Plans to promote participant retention and complete follow-up, including list of any outcome data to be collected for participants who discontinue or deviate	N/A - as this is a feasibility trial retention and lost to follow-up are things we want to know about

For peer review only

		from intervention protocols	
Data management	#19	Plans for data entry, coding, security, and storage, including any related processes to promote data quality (eg, double data entry; range checks for data values). Reference to where details of data management procedures can be found, if not in the protocol	9-10
Statistics: outcomes	#20a	Statistical methods for analysing primary and secondary outcomes. Reference to where other details of the statistical analysis plan can be found, if not in the protocol	9-12
Statistics: additional analyses	#20b	Methods for any additional analyses (eg, subgroup and adjusted analyses)	9-12
Statistics: analysis population and missing data	#20c	Definition of analysis population relating to protocol non-adherence (eg, as randomised analysis), and any statistical methods to handle missing data (eg, multiple imputation)	9-12
**Methods: Monitoring**			
Data monitoring: formal committee	#21a	Composition of data monitoring committee (DMC); summary of its role and reporting structure; statement of whether it is independent from the sponsor and competing interests; and reference to where further details about its charter can be found, if not in the protocol. Alternatively, an explanation of why a DMC is not needed	N/A - formal commitee not needed as this is a minimal risk trail
Data monitoring: interim analysis	#21b	Description of any interim analyses and stopping guidelines, including who will have access to these interim results and make the final decision to terminate the trial	N/A - as above

For peer review only

Harms	#22	Plans for collecting, assessing, reporting, and managing solicited and spontaneously reported adverse events and other unintended effects of trial interventions or trial conduct	N/A
Auditing	#23	Frequency and procedures for auditing trial conduct, if any, and whether the process will be independent from investigators and the sponsor	N/A - as this is a feasiblity study the process of how the intervention works is part of this.
**Ethics and dissemination**			
Research ethics approval	#24	Plans for seeking research ethics committee / institutional review board (REC / IRB) approval	12
Protocol amendments	#25	Plans for communicating important protocol modifications (eg, changes to eligibility criteria, outcomes, analyses) to relevant parties (eg, investigators, REC / IRBs, trial participants, trial registries, journals, regulators)	12
Consent or assent	#26a	Who will obtain informed consent or assent from potential trial participants or authorised surrogates, and how (see Item 32)	7
Consent or assent: ancillary studies	#26b	Additional consent provisions for collection and use of participant data and biological specimens in ancillary studies, if applicable	7
Confidentiality	#27	How personal information about potential and enrolled participants will be collected, shared, and maintained in order to protect confidentiality before, during, and after the trial	7, 9
Declaration of interests	#28	Financial and other competing interests for principal investigators for the overall	12

For peer review only

		trial and each study site	
Data access	#29	Statement of who will have access to the final trial dataset, and disclosure of contractual agreements that limit such access for investigators	9
Ancillary and post trial care	#30	Provisions, if any, for ancillary and post-trial care, and for compensation to those who suffer harm from trial participation	N/A - participants will continue with their usual HIV care
Dissemination policy: trial results	#31a	Plans for investigators and sponsor to communicate trial results to participants, healthcare professionals, the public, and other relevant groups (eg, via publication, reporting in results databases, or other data sharing arrangements), including any publication restrictions	12
Dissemination policy: authorship	#31b	Authorship eligibility guidelines and any intended use of professional writers	13
Dissemination policy: reproducible research	#31c	Plans, if any, for granting public access to the full protocol, participant-level dataset, and statistical code	12
**Appendices**			
Informed consent materials	#32	Model consent form and other related documentation given to participants and authorised surrogates	Attached as additional document
Biological specimens	#33	Plans for collection, laboratory evaluation, and storage of biological specimens for genetic or molecular analysis in the current trial and for future use in ancillary studies, if applicable	N/A - study does not involve biological specimens

Notes:

- 2b: 1-13 - Listed throughout and in trial registration
- 5c: N/A - sponsors/funders to not have a role in study activities

For peer review only

- 11c: N/A - there is nothing for the participants to adhere to apart from attending the Silver Clinic for their appointment, which they will recive a text reminder about.
- 17b: N/A - there is no change to patients HIV care during the trial, therefore if there was an emergency so they would recieve the same care reagrdless of their trial allocation.
- 18b: N/A - as this is a feasibility trial retention and lost to follow-up are things we want to know about
- 21a: N/A - formal commitee not needed as this is a minimal risk trail
- 21b: N/A - as above
- 23: N/A - as this is a feasiblity study the process of how the intervention works is part of this.
- 30: N/A - participants will continue with their usual HIV care
- 32: Attached as additional document
- 33: N/A - study does not involve biological specimens The SPIRIT Explanation and Elaboration paper is distributed under the terms of the Creative Commons Attribution License CC-BY-NC. This checklist was completed on 21. November 2022 using https://www.goodreports.org/, a tool made by the EQUATOR Network in collaboration with Penelope.ai

For peer review only
